# Maize Antifungal Protein AFP1 Elevates Fungal Chitin Levels by Targeting Chitin Deacetylases and Other Glycoproteins

**DOI:** 10.1128/mbio.00093-23

**Published:** 2023-03-22

**Authors:** Lay-Sun Ma, Wei-Lun Tsai, Florensia Ariani Damei, Raviraj M. Kalunke, Meng-Yun Xu, Yu-Han Lin, Hui-Chun Lee

**Affiliations:** a Institute of Plant and Microbial Biology, Academia Sinica, Taipei, Taiwan; Cornell University

**Keywords:** *Ustilago maydis*, chitin deacetylases, CRRSP, DUF26 domain, mannosyltransferase PMT4, antifungal activity, antifungal, chitin, yeast Cda

## Abstract

Pathogenic fungi convert chitin to chitosan to evade plant perception and disarm chitin-triggered immune responses. Whether plants have evolved factors to counteract this evasion mechanism remains obscure. Here, we decipher the mechanism underlying the antifungal activity of maize secretory mannose-binding cysteine-rich receptor-like secreted protein (CRRSP), antifungal protein 1 (AFP1). AFP1 binds to multiple sites on the surface of sporidial cells, filaments, and germinated spores of the biotrophic fungus Ustilago maydis. It inhibits cell growth and budding, as well as spore germination. AFP1 promiscuously interacts with most chitin deacetylases (CDAs) by recognizing the conserved NodB domain to interfere with the enzyme activity. Deletion of *O*-mannosyltransferase 4 decreases protein mannosylation, which correlates with reduced AFP1 binding and antifungal activity, suggesting that AFP1 interacts with mannosylated proteins to exhibit an inhibitory effect. AFP1 also has extended inhibitory activity against Saccharomyces cerevisiae; however, AFP1 did not reduce binding to the double ΔΔ*cda1,2* mutant, suggesting the targets of AFP1 have expanded to other cell surface glycoproteins, probably facilitated by its mannose-binding property. Increasing chitin levels by modulating the activity of cell surface glycoproteins is a universal feature of AFP1 interacting with a broad spectrum of fungi to inhibit their growth.

## INTRODUCTION

Plants safeguard the apoplast environment by deploying cell surface-localized pattern recognition receptors (PRRs) to sense potential danger signals via recognition of pathogen-associated molecular patterns (PAMPs) and plant-derived damaged-associated molecular patterns (DAMPs) (1, 2). These signals alert the plant immune system and lead to the delivery of plant defense molecule blends to the apoplast to ward off pathogenic intruders (3). Adapted pathogens are able to breach this defense barrier by shielding their cell surfaces, sequestering PAMPs, and modifying cell surface glycans to avoid recognition and escape from the host attack at the initial stages of infections (4). In return, plants have evolved new weapons to counteract the fungal camouflage strategies.

The biotrophic fungus Ustilago maydis causes smut disease in maize by inducing large tumors in which the fungal hyphae proliferate and form spores (5). A complex interplay between U. maydis and maize at the interface is mainly governed by an arsenal of fungal effector proteins that are induced in consecutive waves after the fungus contacts and colonizes maize (6). Some functionally characterized effectors have been reported to act in the apoplast to suppress plant immunity and protect fungal hyphae from plant defense molecules. For example, U. maydis Fly1 prevents the hydrolysis of chitin by destabilizing plant chitinases (7), Rsp3 shields the fungal hyphae by blocking the antifungal activity of maize AFP1 (8), and Pit2 and Pep1 inhibit the activities of plant cysteine protease and peroxidases, respectively, to suppress plant immunity (9, 10). Notably, U. maydis also possesses six active chitin deacetylases (CDAs) that convert chitin to chitosan to evade recognition by chitin receptors to promote its virulence and sustain viability (11).

Cysteine-rich receptor-like kinases (CRKs) are a class of transmembrane PRR proteins that feature the domain of unknown function 26 (DUF26; stress antifungal domain PF01657) and harbor a conserved cysteine motif (C-X_8_-C-X_2_-C) in their ectodomain (12, 13). The CRKs have been implicated in plant defense, oxidative salt stress, and salicylic acid responses where the DUF26 domain is also found in some plasmodesmata-localized proteins (PDLPs) (14–17) and cysteine-rich receptor-like secreted proteins (CRRSPs) (12, 13). PDLPs have a similar configuration as the CRKs but lack a kinase domain. They modulate callose deposition in the plasmodesmata to regulate permeability and immunity (17, 18). CRRSPs harboring one or two copies of the DUF26 domain secrete to the apoplast in response to pathogen infection (8, 19). In maize, two CRRSPs, AFP1 and AFP2, are significantly upregulated upon the perception of U. maydis and targeted by the U. maydis effector Rsp3 (8). Silencing of the *AFP1* and paralogous *AFP2* genes in maize increases susceptibility to infection by U. maydis and Colletotrichum graminicola (8), suggesting that they are involved in defense against a broad range of fungal pathogens. Mannose binding-dependent antifungal activities of CRRSPs have so far been demonstrated in maize (antifungal protein 1 [AFP1]) (8) and Ginkgo biloba (GNK2) (20), but not for cotton CRR1, which was shown to stabilize plant chitinases (21). Despite the importance of the DUF26-containing proteins in regulating biotic and abiotic stress responses, their biological function in plant immunity remains unclear, and the CRRSP antifungal mechanism has yet to be elucidated.

Here, we study the maize AFP1 (GenPept accession no. NP_001307755) to provide mechanistic insights into the antifungal activities of a plant CRRSP. We demonstrate that maize AFP1 elevates chitin levels of targeted fungal cells and impairs their viability. This inhibitory effect could be achieved by targeting cell surface glycoproteins, including chitin deacetylases, which are essential for fungal development and virulence. We propose that the interference with the activity of the cell surface glycoproteins regulating the cell wall component biosynthesis in fungi highlights the potential use of AFP1 as an antifungal agent.

## RESULTS

### AFP1 binds to Ustilago maydis sporidia, filaments, and germinating spores.

**FIG 1:**
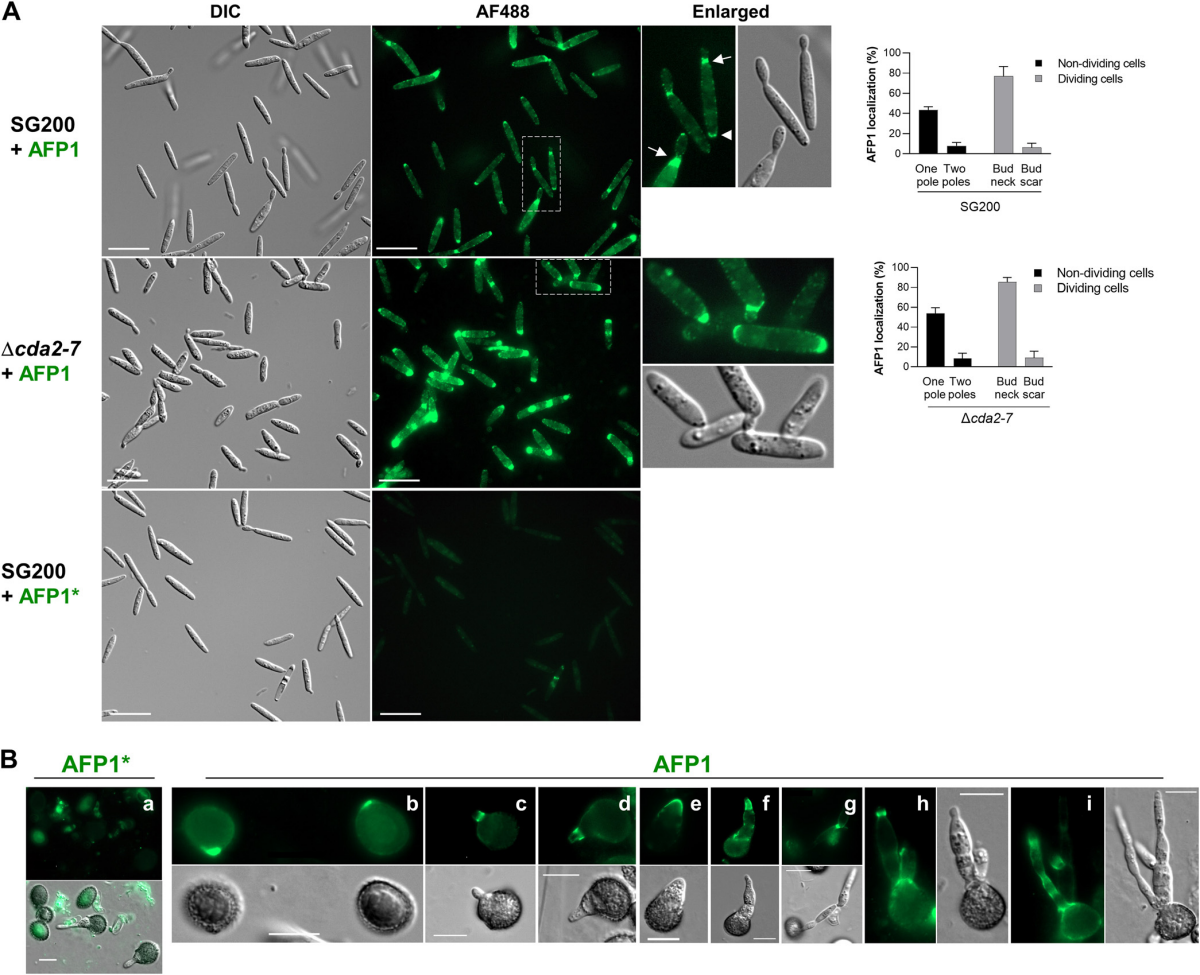
AFP1 binds to multiple sites on Ustilago maydis sporidia and germinating spores. (A) Sporidial cells of indicated strains were incubated with either AFP1-His or AFP1*-His (defects in mannose binding) and immunostained with an anti-His antibody and an AF488-conjugated secondary antibody. Boxed cells are enlarged. Arrows and arrowheads indicate bud necks and scars, respectively. Right panels are quantified results of AFP1 localization. Total dividing cells counted from three independent experiments, 366 (SG200) and 255 (Δ*cda2-7*); nondividing cells, 590 (SG200) and 305 (Δ*cda2-7*). Bars, 20 μm. (B) U. maydis FB1 × FB2-germinated spores were treated with AFP1 and AFP1* proteins. Bars, 10 μm. (B, panels a to i) AFP1-treated germinated spores were immunostained with an anti-His antibody and an AF488-conjugated secondary antibody to visualize AFP1 fluorescence distribution.

We have previously shown that the maize mannose-binding protein AFP1 exhibited antifungal activity toward the U. maydis haploid solopathogenic strain SG200, which does not require a mating partner to complete the life cycle (8, 22). However, the underlying molecular mechanisms remain elusive. To investigate the mode of action of AFP1, we first examined the binding sites of AFP1 on SG200 cells using immunolocalization. Sporidial cells were incubated with purified recombinant epitope-tagged AFP1-His proteins or a mannose binding-deficient variant AFP1*, which contains S34A, R115A, E126A, N144A, Q227A, and E238A mutations in the two mannose-binding sites (8). When these cells were subjected to immunostaining without any permeabilization and fixation, AFP1 was ubiquitously detected as distinct puncta on the cell surface (Fig. 1A). They localized predominantly at the poles of nondividing cells and the bud necks or scars of the dividing cells (Fig. 1A, enlarged panel on the right). They also localized ubiquitously on the surface of SG200 filaments, which was induced on a hydrophobic surface using hydroxyl fatty acids (see Fig. S1A in the supplemental material). AFP1*, on the other hand, did not bind to sporidia and filaments (Fig. 1A and Fig. S1A), suggesting that AFP1 binding to the U. maydis cells could be mannose binding dependent.

10.1128/mbio.00093-23.1 FIG S1 Immunolocalization of AFP1 and CDAs in U. maydis cells. Bars, 20 μm. (A, C, and D) Filaments of indicated strains were induced by hydroxyl fatty acids on the hydrophobic surface, followed by immunostaining using an anti-His antibody or anti-HA antibody and an AF488-conjugated secondary antibody to localize AFP1 or HA_76_Cda1. Arrowheads indicate AFP1 or HA_76_Cda1 fluorescence on hyphal tips. (B) Localization of HA_76_Cda1 and GFP_63_Cda7 in sporidial cells of indicated *CDA* multiple mutants which constitutively expressed CDA proteins under constitutive promoter *otef.* Nonsecreted cytosolic GFP expressed in SG200 was shown. White and orange arrowheads indicate the tip and septa localization of GFP_63_Cda7, respectively. (E) Immunoblot analysis of HA-tagged CDA protein expression. HA-tagged CDA proteins were constitutively expressed in indicated strains, which were grown in YEPSL liquid medium to an OD_600_ of 0.6 to 0.7 before harvesting. Total proteins from cell pellets were prepared and subjected to immunoblot analysis using anti-HA or anti-tubulin antibodies as indicated. Download FIG S1, PDF file, 1.2 MB. Copyright © 2023 Ma et al. 2023 Ma et al. https://creativecommons.org/licenses/by/4.0/ This content is distributed under the terms of the Creative Commons Attribution 4.0 International license.

Spores are the likely propagules by which the infections occur in the field; we therefore tested if AFP1 could also bind to the U. maydis spores to inhibit their germination. Despite some background noise in cell debris and mucilage, no specific fluorescence was detected on the AFP1*-treated germinated spores (Fig. 1B, panel a). AFP1 fluorescence appeared at the bulged regions of spores, presumably the sites of promycelium emergence (Fig. 1B, panel b). AFP1 also bound to the base of promycelium, where it had protruded from spores (Fig. 1B, panels c and d), localized to the tips of the promycelium (Fig. 1B, panels e and f) and the regions between two basidial cells (Fig. 1B, panels g to i). The results illustrate that AFP1 binds to germinating spores at distinct morphological stages during germination, as early as the promycelium’s first protrusion.

### AFP1 blocks spore germination and inhibits cell budding and growth.

**FIG 2:**
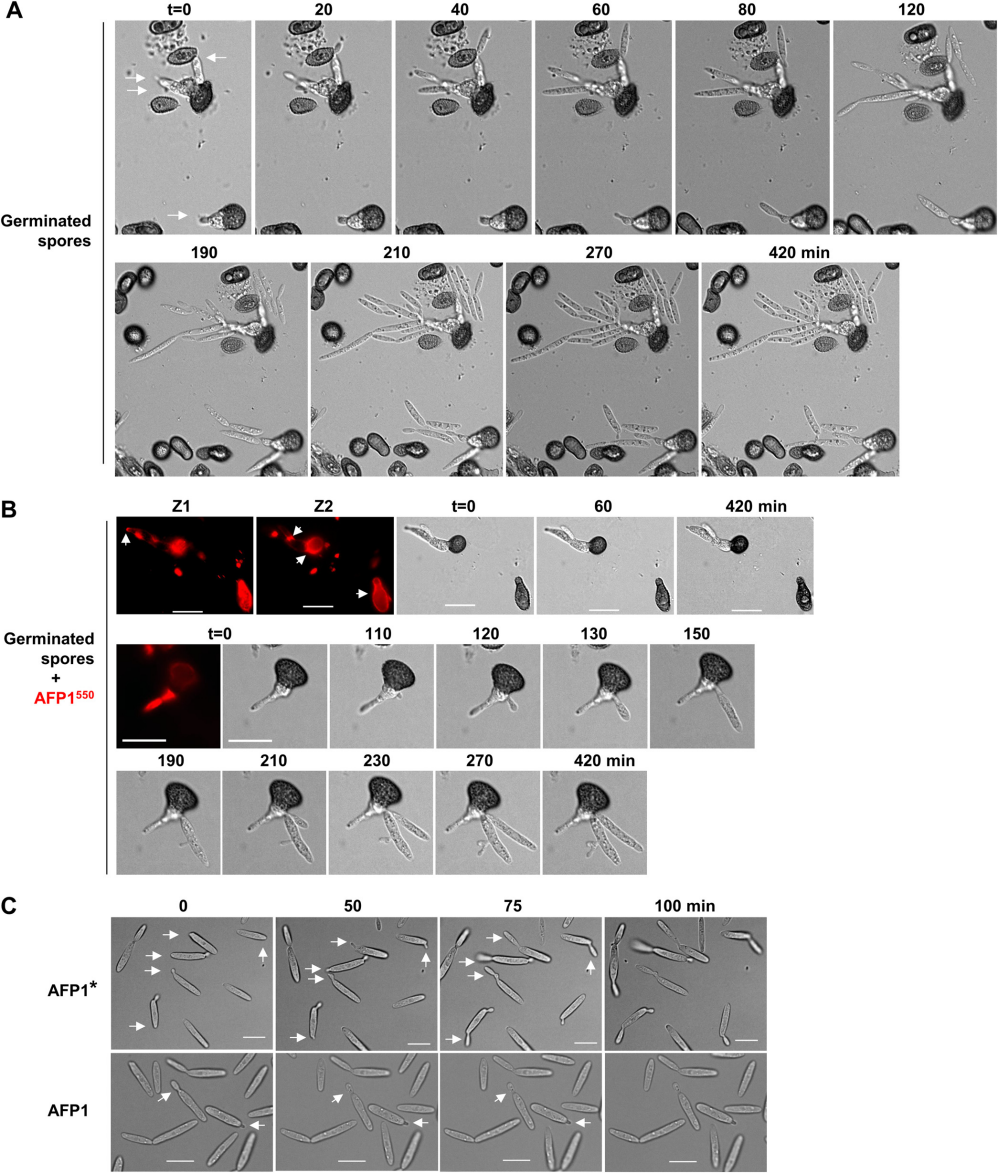
AFP1 inhibits spore germination, cell growth, and budding. (A and B) Images of AFP1^550^-treated and nontreated germinated spores acquired at indicated time points on agar slide containing 2% nutrient. (A) Arrows track basidial cells produced from untreated germinated spores. (B) Arrows indicate the distribution of AFP1^550^ fluorescence on basidial cell/germinated spore captured at different Z-planes. The arrow in Z1 shows AFP1^550^ fluorescence at the tip of the basidial cell; arrows in Z2 indicate AFP1^550^ fluorescence in a region between two basidial cells and in the surroundings of germinated spores. (C) Images of AFP1-treated SG200 sporidial cells acquired at indicated time points. Arrows track cell growth and budding when cells were grown in liquid medium containing 2% nutrients. *t* = 0, the time point after the 4 h incubation with AFP1/AFP1* proteins. Bars, 10 μm.

Owing to asynchronous spore germination and low germination rates in U. maydis (23–26), quantitative inhibitory assays of AFP1 on spore germination are challenging. To dissect the inhibitory activity of AFP1 on spore germination, we performed time-lapse live-cell imaging assays to monitor the germination process of individual spores in the presence or absence of AFP1-His^550^ (Fig. 2A and B). In the absence of AFP1, most spores remained dormant, and only a few promycelia emerged, elongated, produced basidiospores, and then fell off. The germination process slowed down after 5 h and was at a halt in 7 h. The emergence and elongation of promycelia were detected within 20 min and 60 min, depending on the germination stages of spores (Fig. 2A). In contrast, AFP1^550^-bound promycelia stopped elongation (Fig. 2B, top). Promycelia could also abandon the AFP1^550^-bound basidial cells and generate new basidia from alternative sides to produce basidiospores (Fig. 2B, bottom). In this sense, the inhibitory effects of AFP1 were overcome, possibly due to the gradual decrease in the amount of AFP1 protein insufficient to prevent continuous germination. Nevertheless, spore germination was delayed by around 1 h compared to the nontreated spores (Fig. 2A and B).

In sporidial cells, AFP1 inhibited the budding and growth of cells, while the AFP1*-treated cells continued to grow and bud (Fig. 2C). The AFP1-treated cells would eventually die and form large empty compartments after the prolonged incubation. Collectively, the results indicate that AFP1 binding could delay and block spore germination and stop cell growth and budding to exert antifungal activity toward U. maydis.

### Maize AFP1 colocalizes with U. maydis CDAs.

The localization pattern observed for AFP1 in U. maydis cells was similar to that of U. maydis septum- and bud-localized chitin synthases (CHSs) and cell division-localized chitinases (CTSs) (27–29). It also correlated with sites in the cell wall where chitin and chitosan are accessible to antibodies applied to sporidial cells (11). However, no interactions between AFP1 and CHSs or CTSs were found in the yeast two-hybrid (Y2H) assay (Fig. S2A and B). Given that the AFP1 localization bore similarities to those of chitin and chitosan and that chitin-to-chitosan metabolism is important for fungal development and host evasion, we posited that the antifungal activity of AFP1 could be linked with chitin-to-chitosan conversion by targeting chitin deacetylases (CDAs), which are key enzymes in the deacetylation of chitin.

10.1128/mbio.00093-23.2 FIG S2 Yeast two-hybrid assays for the interactions of AFP1 with UmCHSs (chitin synthases), UmCTSs (chitinases), and UmCDAs (chitin deacetylases). Yeast transformants containing two plasmids expressing indicated proteins fused to GAL4 activation domain (AD) or binding domain (BD) without signal peptide were grown on SD-Leu-Trp (LW), SD-Leu-Trp-His-Ade (LWHA), or SD-LWH containing 1 mM of 3-amino-1,2,4-triazole (3-AT) plates for 2 to 3 days. −, empty vector; chorismate mutase (Cmu1) served as the positive control; AD/BD-AFP1, AD/BD-CDA, and AD-CHS/BD served as the negative controls. Similar results were observed in at least two independent experiments. Chs5, UMAG_10277; Chs6, UMAG_10367; Chs7, UMAG_05480; Cts1, UMAG_10419; Cts2, UMAG_02758; Cts3, UMAG_06190; Cts4, UMAG00695. The NodB domain of Cda1 consists of 198 amino acids from 108 to 305. (C, D, and G) Immunoblot analyses of indicated AD and BD fusion protein expression in yeast transformants. Yeast transformants were grown in YPD liquid medium until the OD_600_ reached 0.7 to 0.8. One OD of cell pellet was collected, lysed, and TCA precipitated and analyzed by immunoblots using indicated antibodies. −, empty vector. Two independent clones were selected for immunoblotting analysis. Only clones expressing the expected proteins were used in Y2H analysis. Download FIG S2, PDF file, 0.8 MB. Copyright © 2023 Ma et al. 2023 Ma et al. https://creativecommons.org/licenses/by/4.0/ This content is distributed under the terms of the Creative Commons Attribution 4.0 International license.

**FIG 3:**
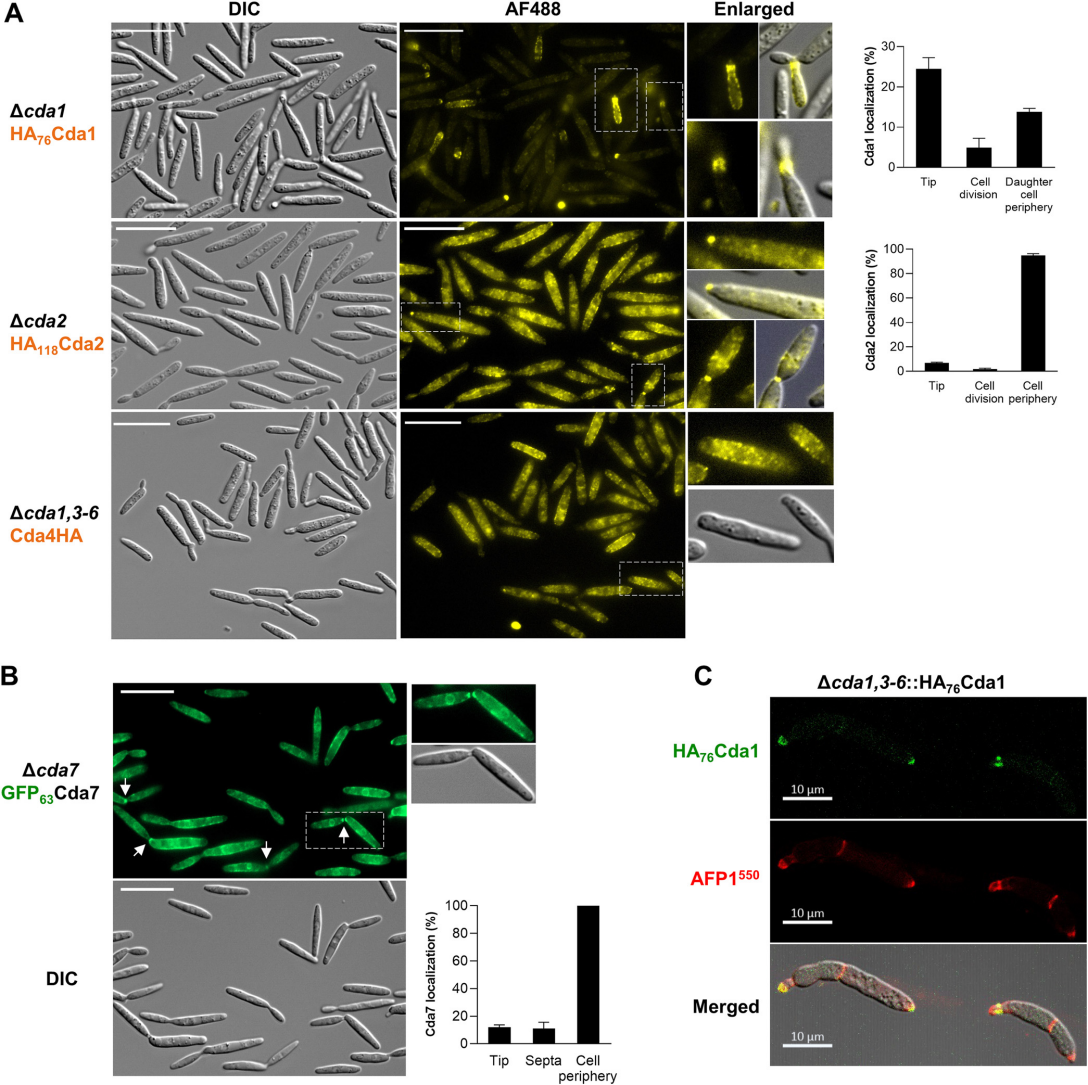
U. maydis CDAs colocalize with AFP1. (A) Indicated *cda* mutants constitutively expressing HA-tagged CDA proteins under the promoter *otef* were immunostained using an anti-HA antibody and an AF488-conjugated secondary antibody to localize CDA proteins. Boxed cells are enlarged. The right panels are quantified results of CDA localization. The total cells counted from three independent experiments are 1,087 (Cda1) and 927 (Cda2). Bars, 20 μm. (B) GFP fluorescence of GFP_63_Cda7 constitutively expressed in Δ*cda7*. Boxed cells are enlarged. Arrows indicate the GFP_63_Cda7 localized at septa. The quantification of Cda7 localization is shown where 660 cells were counted from three independent experiments. Values represent the mean ± standard deviation (SD). (C) Cells of Δ*cda1*,*3-6*::HA_76_Cda1 constitutively expressed HA_76_Cda1 were immunostained to localize Cda1, followed by an incubation with DyLight 550-labeled AFP1-His proteins (AFP1^550^) to visualize the colocalization using confocal microscopy. One representative image from one of the three independent experiments is shown.

The U. maydis genome contains seven *CDA* genes that encode six C-terminal glycosylphosphatidylinositol (GPI)-anchored NodB domain-containing CDAs (Cda1 to Cda3, Cda5 to Cda7), and one secreted Cda4 (Fig. S3A). While *CDA6* is a pseudogene and not expressed (11), the other *CDA* genes are expressed at different levels in the axenic culture of SG200 cells (Fig. S3B). The potential link between AFP1 and CDA proteins in U. maydis pathogenesis was first addressed by examining AFP1 localization in *cda* mutants. Since the deletion of all seven *CDA* genes caused lethality, we examined AFP1-His localization in a Δ*cda2-7* sextuple mutant (11). Surprisingly, instead of detecting weak AFP1 accumulation, we observed an intense AFP1 fluorescence in the Δ*cda2-7* mutant compared to the parental SG200 cells (Fig. 1A). Compensatory upregulation of the *CDA1* gene has been observed in other *cda* mutants such as Δ*cda2-6* and Δ*cda2-5* (Y. S. Rizzi, personal communication), implying that the Cda1 protein may be highly accumulated in the Δ*cda2-7* mutant and targeted by AFP1. To test this hypothesis, we immunolocalized Cda1 to explore the potential colocalization between Cda1 and AFP1. However, we failed to detect an N-terminal hemagglutinin (HA)-tagged Cda1 fusion protein (HA-Cda1) on the cell surface even though the fusion protein was sufficiently expressed (Fig. S1E). This suggests that the Cda1 might undergo posttranslational modification at the N terminus before anchoring to the membrane. To obviate this problem, we inserted the HA epitope at position 76 of Cda1 (HA_76_Cda1; in a disordered region predicted by Phyre2 [30]) and overexpressed in both the Δ*cda1*,*3-6* quintuple (11) and Δ*cda1* mutants. This could prevent interference of the endogenous Cda1 to compete with HA_76_Cda1 for localization. In both backgrounds, HA_76_Cda1 was detected at the growing tips and around the periphery of emerging cells but weakly observed in the periphery of mother cells (Fig. 3A and Fig. S1B). Occasionally, the HA_76_Cda1 puncta were also observed in the cell division zones (Fig. 3A). In the filamentous cells, HA_76_Cda1 localized to the filamentous cell surface and the growing tips (Fig. S1C). The localization pattern of HA_76_Cda1 was comparable to the AFP1 bound to SG200 and Δ*cda1*,*3-6* (Fig. S1A and D).

10.1128/mbio.00093-23.3 FIG S3 *CDA* gene expression in U. maydis and C. graminicola. (A) Schematic drawings of U. maydis CDA proteins with indicated signal peptides, CDA domains, predicted GPI anchors, and serine-threonine (S-T) stretch region. The percentage of S-T enriched in the last 50 amino acid sequences before the GPI anchor is shown. aa, amino acids. Cda1, UMAG_00638; Cda2, UMAG_01143; Cda3, UMAG_11922; Cda4, UMAG_01788; Cda5, UMAG_02019; Cda6, UMAG_05792; Cda7, UMAG_02381. (B) Total RNA was extracted from SG200 cells grown in YEPSL liquid medium and subjected to quantitative RT-PCR. Expression levels of the U. maydis *CDA* gene were normalized relative to the constitutively expressed peptidyl-prolyl isomerase (*PPI*). Values represent the mean ± SD of the three biological replicates. (C) Total RNA extracted from C. graminicola conidial cells grown on agar plates was subjected to semiquantitative RT-PCR. Values were obtained by quantified intensities of PCR bands in the right panel using ImageJ software and normalized relative to the constitutively expressed *ACTIN* gene. Data represent the mean ± SD of the three biological replicates. (D) The percentages of amino acid sequence identity between CDAs of U. maydis and C. graminicola were determined by pairwise sequence alignment calculated using EMBOSS Stretcher (https://www.ebi.ac.uk/Tools/psa/emboss_stretcher/). The orthologs of UmCDAs in CgM2 are predicted by using the server OMA (Orthologous Matrix) (https://omabrowser.org/oma/home/) and highlighted in red. Download FIG S3, PDF file, 0.2 MB. Copyright © 2023 Ma et al. 2023 Ma et al. https://creativecommons.org/licenses/by/4.0/ This content is distributed under the terms of the Creative Commons Attribution 4.0 International license.

In the case of Cda2, the HA_118_Cda2 (an internally tagged) protein was localized to distinct puncta on the cell periphery and at the tips and division zones in Δ*cda2-*HA_118_Cda2 cells (Fig. 3A). Although we could not detect the localization of internally HA-tagged Cda7 (HA_63_Cda7), green fluorescent protein (GFP) fusion proteins (GFP_63_Cda7) accumulated at the cell periphery and the primary and secondary septa of large-budded cells, distinct from the pattern of cytosolic GFP expressed in SG200 (Fig. 3B and Fig. S1B). In contrast, the secreted CDA homolog, Cda4 (C-terminally HA tagged) localized exclusively to the cell periphery when expressed in Δ*cda1*,*3-6* (Fig. 3A). These observations indicate that CDAs display distinct localization patterns and overlap in part with that of AFP1 in U. maydis sporidia and filaments.

We next examined the possible colocalization of AFP1 and Cda1 proteins on the Δ*cda1*,*3-6*::HA_76_Cda1 cells that were subjected to immunostaining before incubation with AFP1^550^. We found overlapping fluorescence between HA_76_Cda1 and AFP1-His^550^ at budding necks and growing tips, suggesting that AFP1 colocalizes with Cda1 in these regions (Fig. 3C). Notably, additional AFP1^550^ fluorescence was also detected at the septa where GFP_63_Cda7 localizes (Fig. S1B). The colocalizations suggest that AFP1 probably interacts with Cda1 and Cda7.

### AFP1 interacts with U. maydis CDAs.

**FIG 4:**
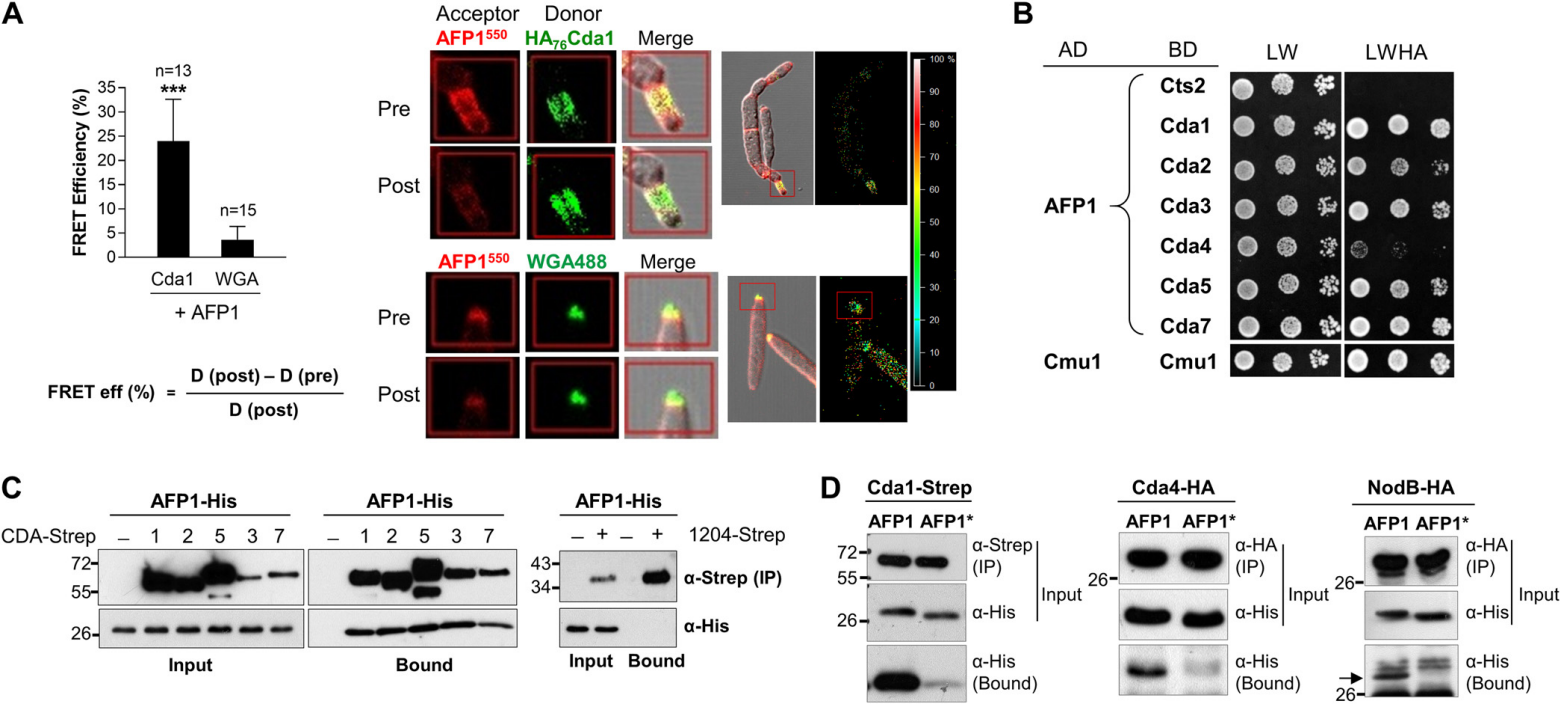
AFP1 interacts with UmCDAs. (A) The Δ*cda1*,*3-6*::HA_76_Cda1 strain was immunostained to localize Cda1 or stained with WGA-AF488 to locate chitin prior to an incubation with DyLight 550-labeled AFP1 (AFP1^550^). FRET efficiency was calculated using the indicated equation. D, donor. The number of spots from three independent experiments used in the analysis is indicated above columns. Values represent the mean ± SD. (A, Left) Representative images of cells displaying fluorescence of AFP1^550^ and HA_76_Cda1/WGA-AF488 taken before and after the photobleaching of acceptor AFP1^550^ are shown. (A, Right) Cell images with overlapped fluorescence of HA_76_Cda1/WGA-AF488 and AFP1^550^ and the signal intensity in overlapping regions are shown. ***, significant differences in FRET efficiency of HA_76_Cda1 and chitin staining determined by a two-tailed Student's *t* test, *P* < 0.001. (B) Yeast transformants containing two plasmids expressing indicated GAL4 activation domain (AD) or binding domain (BD) fusion proteins were grown on SD-Leu-Trp (LW) and SD-Leu-Trp-His-Ade (LWHA) plates for 3 days. Self-interaction of chorismate mutase (Cmu1) served as the positive control and AD-AFP1/BD-Cts2 (chitinase 2) as the negative control. Additional negative controls (AD/BD-CDAs and AD-AFP1/BD) are shown in Fig. S2D and E in the supplemental material. Similar results were observed in at least two independent experiments. (C) *In vitro* pulldown assay of CDA-Strep and AFP1-His proteins. Indicated C-terminal Strep-tagged CDA or UMAG1204 (non-CDA mannoprotein) proteins from culture supernatants of Δ*cda1*,*3-6* constitutively expressing Cda1, Cda3, or Cda5-Strep, Δ*cda2-7* constitutively expressing Cda2 or Cda7-Strep, and SG200 constitutively expressing 1204-Strep were immobilized on Strep-Tactin agarose beads and served as baits to pull down AFP1. (D) Cda1-Strep and Cda4HA proteins were immunoprecipitated from the culture supernatant of Δ*cda1*,*3-6*::Cda1Strep and Δ*cda1*,*3-6*::Cda4HA using Strep-Tactin and HA agarose beads, respectively, before being incubated with AFP1 and AFP1*. NodB (residues 108 to 305 fused to the signal peptide of Cda1; SP-NodBHA) was immunoprecipitated from the cell lysate of Δ*cda1*::NodBHA. Arrow indicates AFP1. One representative blot from at least two independent experiments is shown.

To examine the interaction of Cda1-AFP1 in intact cells, we measured acceptor photobleaching fluorescence resonance energy transfer (FRET) efficiency (31–33). The FRET efficiency of HA_76_Cda1 after bleaching AFP1-His^550^ increased significantly by an average of 23%, compared to 4% in the negative control, chitin stained by wheat germ agglutinin (WGA)-Alexa Fluor 488 (AF488) (Fig. 4A), suggesting that Cda1 and AFP1 are within close proximity as interacting partners in U. maydis.

The colocalization of AFP1 and CDAs prompted us to investigate whether AFP1 interacts with other CDAs. Y2H analysis indicated that AFP1 interacted with most CDAs but showed a weaker association with Cda4 despite all tested CDAs expressed at similar levels (Fig. 4B and Fig. S2C). In contrast to AFP1, AFP1* lost interaction with Cda2, Cda4, and Cda5 proteins but retained interaction with Cda1, Cda3, and Cda7 (Fig. S2F and G). Increasing the selection stringency with 3-amino-1,2,4-triazole (3-AT) demonstrated that Cda1 had a relatively stronger affinity than Cda7 for AFP1 and AFP1*, but Cda7 has a comparatively weaker affinity to AFP1* than AFP1 (Fig. S2H and I). Furthermore, the NodB domain of Cda1 was sufficient to mediate the AFP1-Cda1 interaction in the Y2H (Fig. S2J).

We next validated the specificity of the AFP1-CDA interaction by *in vitro* pulldown assays. The GPI anchor-deleted and C-terminally fused streptavidin-tagged Cda1, Cda2, Cda3, Cda5, and Cda7 (Cda-Strep) proteins were all able to pull down AFP1, whereas buffer controls and a non-CDA mannosylated protein (UMAG1204) that localized at the cell division zone failed to do so (Fig. 4C; Fig. S4 and S5A). Under high-salt conditions (0.5 M NaCl), Cda4, Cda1, and the NodB domain of Cda1 effectively pulled down AFP1 but not AFP1* (Fig. 4D). This suggests that AFP1 interacts with Cda1 through the NodB domain and that the two DUF26 domains of AFP1 are required for mannose and NodB binding. Altogether, AFP1 promiscuously interacts with all six U. maydis CDAs, and the mannose binding of AFP1 can facilitate the interactions.

10.1128/mbio.00093-23.4 FIG S4 Localization and ConA blot analysis of UMAG1204. (A) Immunolocalization of non-CDA mannoprotein UMAG1204HA expressing in SG200 cells under constitute promoter *otef*. Bar, 20 μm. (B) Mannosylation of UMAG1204 by ConA blot analysis. Tagged proteins were constitutively expressed in indicated strains and immunoprecipitated from culture supernatants, separated on SDS-PAGE, incubated with and without concanavalin A (ConA), and blotted against anti-ConA and anti-HA antibodies. Arrow indicates 1204HA proteins. Download FIG S4, PDF file, 0.2 MB. Copyright © 2023 Ma et al. 2023 Ma et al. https://creativecommons.org/licenses/by/4.0/ This content is distributed under the terms of the Creative Commons Attribution 4.0 International license.

10.1128/mbio.00093-23.5 FIG S5 Prediction of *O*- and *N*-glycosylation sites in UmCHSs, UmCTSs, CDAs, and UMAG1204. (A to C) *O*-mannosylation sites were predicted by the online NetOGlyc server (https://services.healthtech.dtu.dk/service.php?NetOGlyc-4.0). The cutoff value was set at ≥0.7. (A) Putative *O*-mannosylation sites for ScCDAs, CgCDAs, and UMAG1204. (B) Putative *O*-mannosylation sites for U. maydis UmCDAs. (C) Putative *O*-mannosylation sites for U. maydis UmCHSs and UmCTSs. No mannosylation site was predicted for UmCts2. (D) Putative *N*-glycosylation sites of CDAs, UmCHSs, UmCTSs, and UMAG1204 proteins predicted by the online NetNGlyc server (https://services.healthtech.dtu.dk/service.php?NetNGlyc-1.0). No *N*-glycosylation site was predicted for UmCda4 and C. graminicola GLRC_0386. Download FIG S5, PDF file, 0.2 MB. Copyright © 2023 Ma et al. 2023 Ma et al. https://creativecommons.org/licenses/by/4.0/ This content is distributed under the terms of the Creative Commons Attribution 4.0 International license.

### U. maydis CDAs are mannosylated proteins.

**FIG 5:**
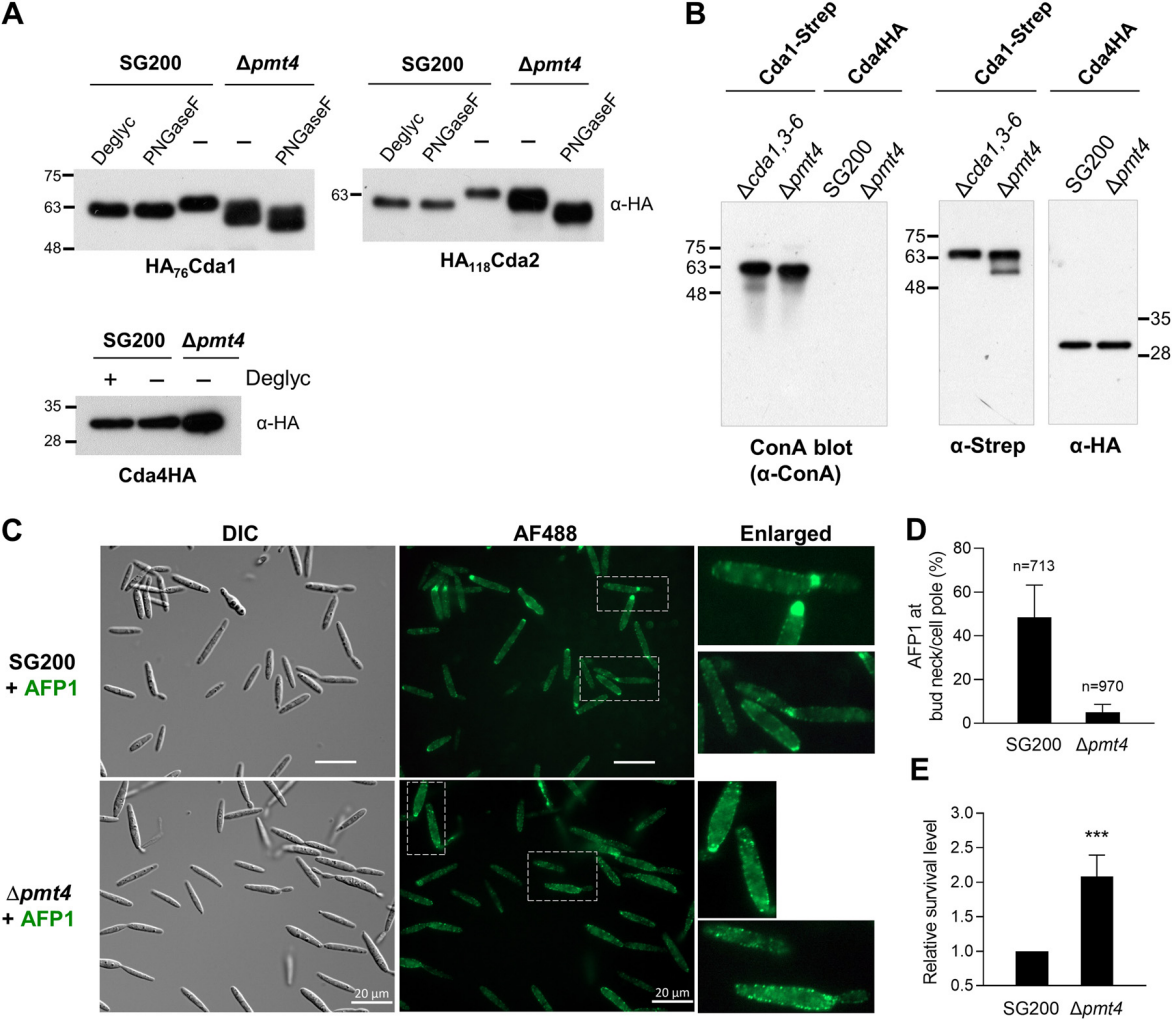
Cda1 and Cd2 are partly mannosylated by Pmt4. (A) Deglycosylation of CDAs. Total proteins from the cell pellets of SG200/Δ*pmt4* constitutively expressing indicated CDAs were treated with (+) or without (−) deglycosylation enzyme mix (Deglyc) or PNGase F according to the manufacturer’s protocol. The migration pattern of CDA proteins was analyzed by immunoblotting using an anti-HA antibody. (B) Detection of Cda1 mannosylation by lectin blotting. Cda1-Strep and Cda4HA proteins were immunoprecipitated from the culture supernatant of indicated strains and separated on SDS-PAGE. PVDF membranes were incubated with or without concanavalin A (ConA), followed by immunoblotting using anti-ConA, anti-HA, or anti-Strep antibodies. (C) Sporidial cells of indicated strains were incubated with AFP1-His, followed by immunostaining, and visualized by fluorescence microscopy. Bars, 20 μm. (D) AFP1 reduces binding to Δ*pmt4*. The total number of cells (*n*) from three independent experiments in experiment C is indicated above the columns. The percentage of cells with AFP1 binding at bud necks/cell poles was calculated. Values represent the mean ± SD of three experiments. (E) Deletion of *PMT4* increases cell survival. SG200 and Δ*pmt4* cells incubated with AFP1 and AFP1* were diluted and plated. The number of viable cells treated with AFP1* was set to 1 to normalize the number of viable cells treated with AFP1. The survival number of Δ*pmt4* cells was relative to SG200 surviving cells. Values represent the mean ± SD of four independent experiments. Asterisks indicate significant differences between two strains determined by a two-tailed Student's *t* test. ***, *P* < 0.001.

We next investigated the functional relevance of the mannose-binding capacity of AFP1 for interacting with CDAs. The NetOGlyc 4.0 server (34) with cutoff values of ≥0.7 predicted at least 18 putative *O*-mannosylation sites on each CDA, with the exception that Cda4 bears only six putative sites (Fig. S5B). The putative sites are distributed mainly at the C-terminal Ser-Thr stretch region of CDAs, which Cda4 lacks (Fig. S3A). By the prediction of the NetNGlyc 1.0 server (35), most CDAs contain putative *N*-linked glycosylation sites except for Cda4 (Fig. S5D). To validate these predictions, we examined migration patterns of CDA proteins after incubation with deglycosylation enzyme mixture (which removes *O*- and *N*-glycans) and peptide-*N*-glycosidase F (PNGase F; specifically removes *N*-glycans). For Cda1 and Cda2, but not Cda4, altered migration patterns were observed that were similar in both treatments (Fig. 5A). This illustrates that only *N*-glycans of CDAs could be removed, providing evidence that Cda1 and Cda2 are *N*-glycosylated.

The *O*-mannosyltransferase 4 (Pmt4) has been reported to *O*-mannosylate the Ser-Thr stretch in membrane-associated proteins (36, 37) and is required for U. maydis early infection-related development (38). We therefore investigated if Cda1, Cda2, and Cda4 proteins are specifically modified with mannan by Pmt4. When expressed in the Δ*pmt4* background, Cda1 and Cda2 proteins exhibited smeary and faster migration patterns and increased further after PNGase F treatment (Fig. 5A and Fig. S6A). In contrast, migration patterns of Cda4 were similar regardless of the genetic background or enzymatic treatment (Fig. 5A). This suggests that Cda1 and Cda2, but not Cda4, are substrates for *O*-mannosylation by Pmt4 in addition to their *N*-linked glycosylation.

10.1128/mbio.00093-23.6 FIG S6 Analysis of UmCDA mannosylation and localization and pH-dependent killing activity of AFP1. (A) Total proteins from the cell pellet of indicated strains expressing respective CDA proteins under the constitutive promoter *otef* were collected and separated on SDS-PAGE. The migration patterns of CDA proteins were analyzed by immunoblotting using an anti-HA antibody. (B) ConA recognized mannan of Cda2. Cda2-Strep and Cda4HA were constitutively expressed in indicated strains, immunoprecipitated from the culture supernatants, and analyzed by blotting with ConA and by immunoblotting using anti-ConA, anti-HA, and anti-Strep antibodies. *, nonspecific protein bands. (C) Effect of pH on AFP1 killing activity. SG200 cells were incubated with AFP1 or AFP1* in either PBS buffer (pH 7.2) or MES buffer (pH 5.5) for 4 h. The number of cells that survived in the AFP1 treatment was relative to the number of AFP1*-treated surviving cells, which was set as 1. Values represent the mean ± SD of three independent experiments. Asterisks indicate significant differences between the two treatments determined by a two-tailed Student’s *t* test. ***, *P* < 0.001. (D) Immunolocalization of HA_76_Cda and HA_118_Cda2. Cells expressing HA-tagged CDA proteins under constitutive promoter *otef* in indicated strains were subjected to immunostaining using an anti-HA antibody and an AF488-conjugated secondary antibody to localize CDA proteins. Bars, 20 μm. (D, Right) SG200 cells subjected to immunostaining using an anti-HA antibody, and AF488-conjugated secondary antibody served as negative control. Download FIG S6, PDF file, 0.6 MB. Copyright © 2023 Ma et al. 2023 Ma et al. https://creativecommons.org/licenses/by/4.0/ This content is distributed under the terms of the Creative Commons Attribution 4.0 International license.

To further examine the mannosylation of CDAs, we immunoprecipitated GPI anchor-deleted CDA and Cda4 proteins from culture supernatants and assessed them through blotting with mannose-binding concanavalin A (ConA). Mannose structures were evident on the Cda1 and Cda2 but not on Cda4, which were detected by antibodies against their respective protein tags (Fig. 5B and Fig. S6B). The results support that Cda1 and Cda2 are mannosylated proteins. No noticeable migration difference and undetectable Cda4 might be due to the degree of mannosylation on the protein falling below the detection sensitivity.

We next investigated if the binding efficiency of AFP1 is compromised in the Δ*pmt4* background. We anticipated that if binding to the mannan structure of CDAs could facilitate the AFP1-CDA interaction, AFP1 most probably would reduce binding to cells of Δ*pmt4.* Indeed, in Δ*pmt4*, the prominent AFP1 association with the bud necks and scars was largely diminished, whereas the punctate localization of AFP1 on the cell periphery became more evident (Fig. 5C and D). Although AFP1 could bind to SG200 cells at a basic pH (Fig. 1 and Fig. 5C), we only observed a dramatic killing effect of AFP1 at acidic pH (Fig. S6C). We thus examined the effect of AFP1 killing on Δ*pmt4* cells under the acidic condition. Reduced AFP1 binding to the Δ*pmt4* mutant was correlated with more than 2-fold-higher cell viability than SG200 (Fig. 5E), highlighting the importance of mannose binding to the antifungal activity of AFP1. Since Cda1 and Cda2 localization was comparable in SG200 and Δ*pmt4* (Fig. S6D), the reduced binding of AFP1 to the site of cell division of Δ*pmt4* is likely due to decreased interactions with either Cda1 and Cda2 proteins or other glycoproteins which have defects in the degree or pattern of mannosylation in the absence of Pmt4. Altogether, our findings suggest the functional importance of mannose binding for AFP1 in interactions with the cell surface-mannosylated CDAs and probably other glycoproteins.

### AFP1 interferes with chitin-to-chitosan metabolism.

**FIG 6:**
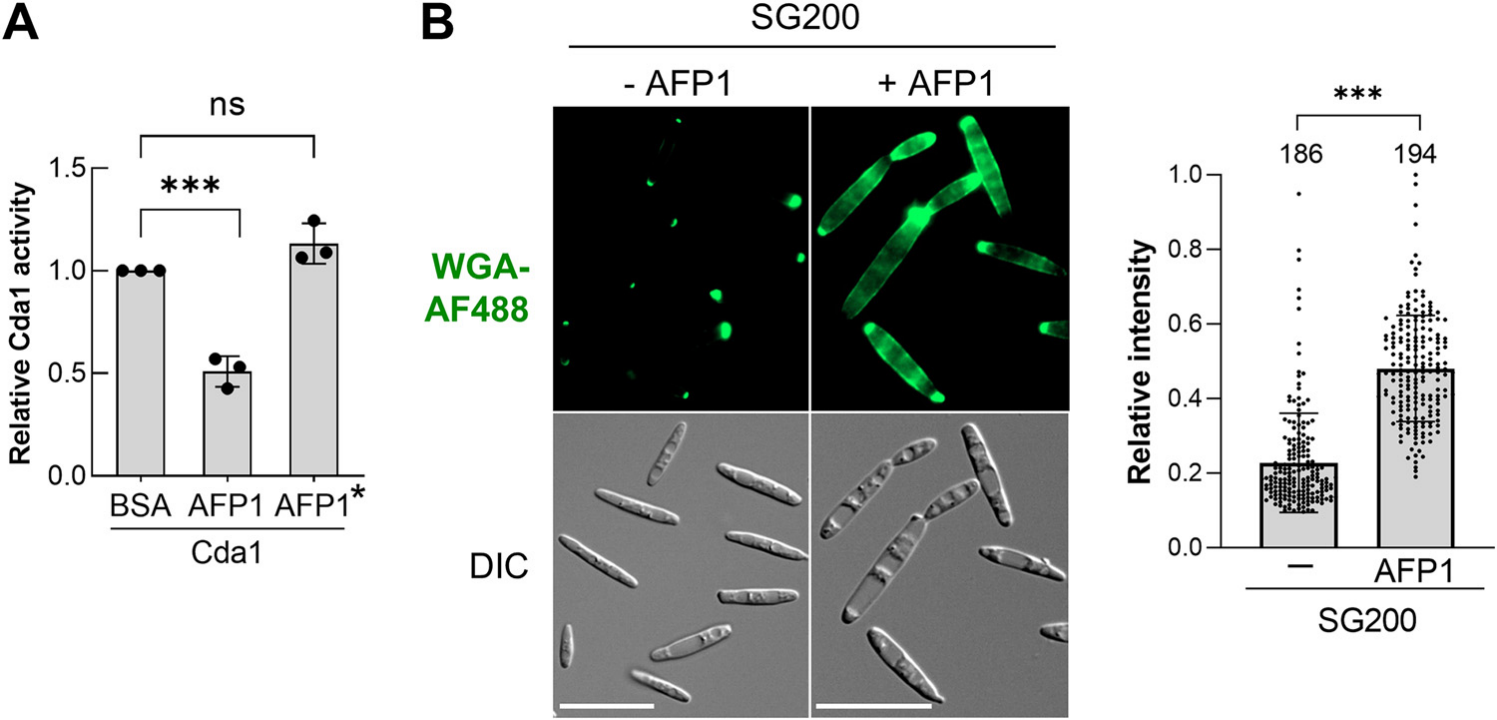
AFP1 interferes with Cda1 activity and increases chitin level. (A) Secreted Cda1-Strep proteins from the Δ*cda1*,*3-6*::Cda1Strep strain were immobilized on Strep-Tactin agarose beads and treated with AFP1, AFP1*, or BSA proteins before incubation with GlcNAc_5_ (A5) substrates. Cda1 activities in the presence of AFP1/AFP1* are relative to BSA control, which is set as 1. Three independent batches of culture supernatant were collected to perform a CDA activity assay. Values represent the mean ± SD of three independent experiments (3 technical replicates per experiment). Significant differences in CDA activity compared to BSA control were determined by a two-tailed Student's *t* test. ***, *P = *0.0003; ns, not significant. Representative silver-stained gel showing a similar amount of Cda1Strep and AFP1 proteins loaded in each reaction can be found in Fig. S7C in the supplemental material. (B) WGA488-chitin staining in AFP1-treated cells. (B, Left) AFP1-treated or untreated SG200 cells were stained with WGA-AF488. (B, Right) Fluorescent intensities of WGA-stained cells were determined using ImageJ and normalized to the highest intensity of a cell in the same experiment, which is set as 1. The total number of quantified cells from three independent experiments is indicated above the respective boxes. Asterisks indicate significant differences between AFP1-treated and untreated cells determined by a two-tailed Student's *t* test. ***, *P* < 0.001. Bars, 20 μm.

As CDAs are one of the AFP1 targets, AFP1 might influence U. maydis cell wall chitin levels. Due to the instability of eluted Cda1-Strep proteins, Cda1-Strep proteins were immobilized on beads and incubated with AFP1, followed by incubation with penta-*N*-acetylchitopentaose substrate (GlcNAc_5_ [A5]). The relative amount of released acetate was significantly decreased when the Cda1-Strep proteins were incubated with AFP1-His, but not in the case of AFP1*-His (Fig. 6A and Fig. S7A). Our results suggest that binding of the AFP1 to Cda1 reduces the deacetylase activity of CDA *in vitro*.

10.1128/mbio.00093-23.7 FIG S7 Inhibition of UmCda1 activity by AFP1 and chitin staining analysis. (A) UmCda1-Strep proteins bound on beads were preincubated with different amounts of AFP1-His, ranging from 0 to 10 μg (1 μM) AFP1 or 10 μg of AFP1*-His for 4 h at 25°C before the incubation with GlcNAc_5_ for additional 2 h. The hydrolytic activity towards GlcNAc_5_ was determined. Data are means ± SDs of three independent determinations. UmCda1 activities were relative to the activity at 0 μg of AFP1 (1 μM of BSA), which was set as 1. (B) Cells of indicated strains in the exponential phase were stained with WGA-AF488 to detect chitin. Bars, 20 μm. (C) Representative silver staining gel image of indicated protein loading in each reaction for CDA activity shown in Fig. 6A. Download FIG S7, PDF file, 0.3 MB. Copyright © 2023 Ma et al. 2023 Ma et al. https://creativecommons.org/licenses/by/4.0/ This content is distributed under the terms of the Creative Commons Attribution 4.0 International license.

We next stained the chitin of AFP1-treated cells using the WGA-488 probe. The AFP1-treated cells strongly increased chitin staining and formed large empty compartments (Fig. 6B). A chitin-stained zebra-crossing pattern observed on the AFP1-treated cells was in line with the Δ*cda2-7* (Fig. S7B) and other *cda* mutants reported previously (11). The result suggests that AFP1 binding at least interferes with the activity of CDAs, which, in turn, affects fungal chitin metabolism.

### AFP1 targets other fungal cell surface glycoproteins.

To investigate if AFP1 interacts with CDAs from other fungi and extends the inhibitory effects toward them, we examined the binding of AFP1 to the maize pathogen Colletotrichum graminicola CgM2, which had increased virulence in *AFP1*- *and AFP2*-silenced plants (8). Six of the seven *CgCDA* genes were expressed in the CgM2 conidial cells (Fig. S3C). They have a relatively low percentage (18 to 30%) of protein identity to U. maydis UmCDAs (Fig. S3D). The two putative glycosylated highly expressed C. graminicola GLRG_7915 and GLRG_386 proteins, which are the orthologs respective to UmCda5 and UmCda4, interacted with AFP1 in the Y2H assay (Fig. S8A). AFP1 fluorescence was detected on the CgM2 conidial cell poles (Fig. S8A), suggesting that C. graminicola is also targeted by maize AFP1.

10.1128/mbio.00093-23.8 FIG S8 AFP1 extends the inhibitory effect towards C. graminicola and S. cerevisiae. (A, B) C. graminicola and S. cerevisiae cells were treated with AFP1 and AFP1* proteins, followed by immunostaining to detect the localization of AFP1. Bars, 10 μm. (A, E) Yeast two-hybrid assays to detect the interactions of AFP1 and CDA proteins of C. graminicola (A) or S. cerevisiae (E). Yeast transformants containing two plasmids expressing the indicated protein fusion to the GAL4 activation domain (AD) or binding domain (BD) were grown on SD-LW for growth control and on SD-LWHA or LWH plates containing 10 mM of 3-AT to assess protein interaction. −, empty vector. Self-interaction of chorismate mutase (Cmu1) served as a positive control, and AD-AFP1/BD and AD/BD-CDA were used as negative controls. Similar results were observed in at least two independent experiments. (C) qRT-PCR analysis of S. cerevisiae *CDA* genes. Total RNA extracted from BY4742 cells after the 4 h-incubation in YPD medium, 1× PBS buffer (pH 7.2), and 10 mM MES (pH5.5) buffer was subjected to qRT-PCR analysis. Expression levels of *CDA* genes are normalized to the *ACTIN* gene and relative to the expression level of *CDA* in YPD, which is set to 1. Data represent the mean ± SD of the three biological replicates. (D) S. cerevisiae Δ*cda1*::HA_75_ScCda1 cells constitutively expressing HA_75_ScCda1 from the *gpd* promoter were immunostained to localize ScCda1 or stained with WGA-AF488 to locate chitin, prior to incubation with DyLight 550-labeled AFP1 (AFP1^550^). The number of spots from three independent experiments used in the analysis is indicated above columns. Values represent the mean ± SD. Representative images of cells displaying fluorescence of AFP1^550^ and HA_75_ScCda1/WGA-AF488 taken before and after photobleaching AFP1^550^ were shown. ns, no significant differences in FRET efficiency between HA_75_ScCda1 and WGA488-stained chitin determined by a two-tailed Student’s *t* test. Download FIG S8, PDF file, 0.4 MB. Copyright © 2023 Ma et al. 2023 Ma et al. https://creativecommons.org/licenses/by/4.0/ This content is distributed under the terms of the Creative Commons Attribution 4.0 International license.

**FIG 7:**
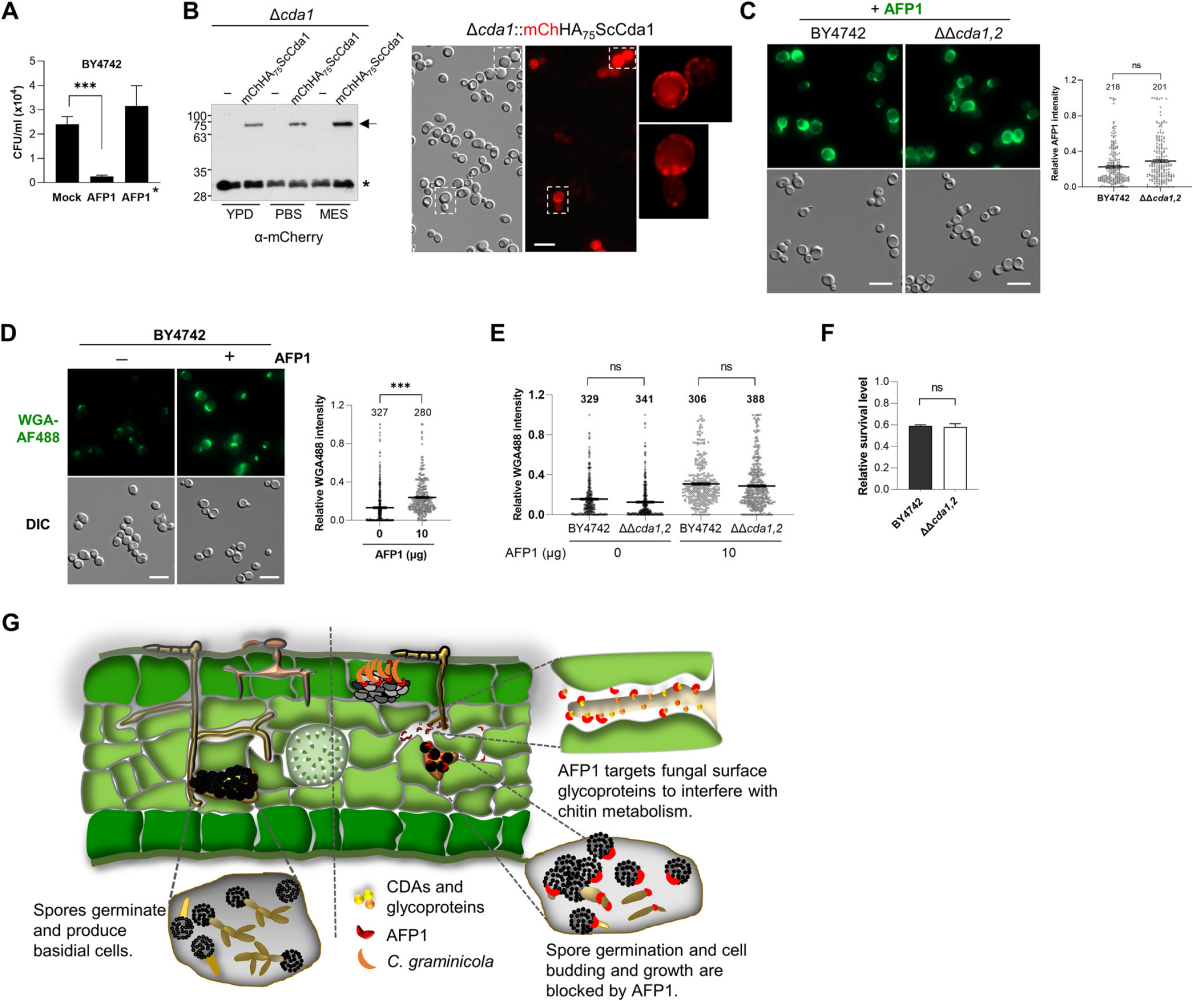
AFP1 targets S. cerevisiae cell surface glycoproteins to increase chitin level and impair cell viability. (A) AFP1 inhibits S. cerevisiae cell growth. BY4742 cells were incubated with 20 μg AFP1, AFP1*, and mock (buffer) for 4 h, serially diluted, and plated on YPD agar. Colony-forming unit (CFU) of S. cerevisiae cells growing on agar plates were quantified. Values represent the mean ± SD of three independent experiments. (B) Expression and localization of ScCda1 in S. cerevisiae. (B, Left) Δ*cda1*::mCherryHA_75_ScCda1 cells expressing mCherry-HA (mChHA)-ScCda1 fusion protein under the native promoter were cultured in liquid YPD medium, MES, and PBS buffer for 4 h. Total proteins from one OD_600_ of cell pellets were extracted and subjected to immunoblotting. −, empty vector; *, nonspecific bands. Arrows indicate full-length ScCda1 fusion protein. (B, Right) Fluorescence of mCherryHA_75_ScCda1 was visualized in the indicated cells cultured in PBS buffer. Similar localization patterns of the ScCda1 fusion protein were observed for the indicated strain growth in liquid YPD medium and MES buffer. Boxed cells are enlarged to visualize punctate foci of ScCda1 proteins. Bars, 10 μm. (C) AFP1 binds to S. cerevisiae ΔΔ*cda1*,*2*. Wild-type BY4742 and ΔΔ*cda1*,*2* cells were treated with AFP1 proteins in parallel before immunostaining. (D and E) WGA488-chitin staining of indicated S. cerevisiae cells with or without AFP1 treatment. (C to E) Intensities of AFP1 and WGA were determined using ImageJ and normalized to the highest intensity of a single cell in the same experiment, which is set as 1. The number of quantified cells from three independent experiments is indicated above the respective boxes. ***, *P < *0.001, significant differences between treatments/strains determined by a two-tailed Student's *t* test; ns, no significant differences. (F) Indicated cells were treated with 10 μg AFP1 and mock in parallel. Surviving cells from each indicated strain were relative to respective mock control, which was set as 1. Values represent the mean ± SD of three independent experiments. (G) Upon pathogen invasion, plants deliver AFP1 proteins to the apoplast to stop fungal colonization. AFP1 targets and interferes with the activity of glycoproteins, including CDAs, which have regulatory roles in fungal cell wall assembly or chitin metabolism, to impair fungal cell development and virulence (11). By acting on fungal glycoproteins, AFP1 prevents spore germination and inhibits cell budding and growth, ultimately leading to fungal cell death and blocking fungal invasion.

Besides targeting C. graminicola, Saccharomyces cerevisiae is also inhibited by AFP1. AFP1 bound to the cell periphery and cell division zone and reduced the viability of S. cerevisiae cells (Fig. S8B and Fig. 7A). S. cerevisiae encodes two *ScCDA* genes expressed during sporulation that are not essential for cell viability in the vegetative stage (39). We therefore investigated the binding of AFP1 to the S. cerevisiae double deletion mutant ΔΔ*cda1*,*2* to explore if AFP1 has additional cell surface targets. Reverse transcription-quantitative PCR (qRT-PCR) analysis showed that both *ScCDA1* and *ScCDA2* genes were highly induced in MES (morpholineethanesulfonic acid) and phosphate-buffered saline (PBS) buffers (Fig. S8C). This result is in line with a previous report that nonmetabolizable buffers and low pH induce *ScCDA1* and *ScCDA2* gene expression and support S. cerevisiae sporulation (40). Although *ScCDA2* had a higher expression than *ScCDA1*, we could not detect the expression of ScCda2 fusion proteins (ScCda2HA, HA_66_ScCda2, or mCherry_66_ScCda2) from the native promoter when cells were cultured in yeast extract-peptone-dextrose (YPD) medium or assay buffer. In contrast, using a similar approach and growth conditions, ScCda1 fusion protein (mCherryHA_75_ScCda1) was detected using immunoblotting and fluorescence microscopy (Fig. 7B). However, only a few cells had punctate mCherry fluorescent foci on the surface, and most cells had relatively weak or undetectable signals (Fig. 7B). This demonstrates that ScCda1 is expressed and localized to the cell surface of vegetative haploid cells.

Although ScCda1 and AFP1 colocalized on the cell periphery and cell division sites, no interaction between AFP1 and ScCda1 was found using an acceptor photobleaching assay (Fig. S8D). In the Y2H assay, the AFP1-ScCda1 interaction could not be determined due to high false-positive background (Fig. S8E). Deletion of the Sc*CDA1* and Sc*CDA2* genes did not abolish the AFP1 binding to ΔΔ*cda1*,*2* cells, and AFP1 signals on both wild-type BY4742 and ΔΔ*cda1*,*2* cells were comparable (Fig. 7C). These results suggest that AFP1 interacts with other glycoproteins on the surface of S. cerevisiae cells.

Consistently, AFP1 binding increased chitin levels of S. cerevisiae cells (Fig. 7D). This highlights that elevated chitin level of targeted cells is a feature of AFP1 inhibition. By comparing survival cell numbers and chitin levels of AFP1-treated wild-type and ΔΔ*cda1*,*2* cells, there was no significant difference between the two strains (Fig. 7E and F). These findings suggest that AFP1 antagonizes a broad spectrum of fungi to modulate chitin levels via targeting glycoproteins, including CDAs.

## DISCUSSION

In this study, we demonstrate that AFP1 confers antifungal activity by targeting cell surface glycoproteins to increase chitin levels and arrest fungal cell growth. Given the importance of chitin and chitosan in providing structural stability to fungal cell walls, determining fungal virulence, and as scaffolds for attachment to other wall components (11, 41–44), their biosynthesis needs to be spatially and temporally regulated in response to environmental changes. AFP1 targets fungal CDAs and other glycoproteins associated with the chitin metabolism or cell wall architecture, providing an effective strategy to inhibit fungal colonization. This manifests as inhibition of fungal cell growth and spore germination, activation of chitin-triggered plant immunity, and, ultimately, protection against fungal invasion (Fig. 7G). Since the U. maydis effector Rsp3 counteracts the activity of AFP1 (8), the window for AFP1 to act is likely during early infection when the *RSP3* gene is not expressed, i.e., prior to appressorium formation. This will permit AFP1 to inhibit spore germination and emerging sporidia while avoiding Rsp3.

AFP1 highly accumulates at the site of cell division and, to a lesser extent, at the cell periphery, where most CDAs and glycoproteins localize. The outer wall of fungal hyphae covered by the *N*-mannans of glycoproteins, especially the cell wall of Candida albicans (45), has been proposed to function as a β-glucan barrier to protect the inner cell walls, where GPI-anchored cell proteins and glycoproteins are located. In addition to the protection provided by the U. maydis effector Rsp3, the *N*-mannan layer of fungal hyphae may also prevent AFP1 from reaching its targets in the inner cell walls. We propose that the cell walls of actively growing cellular regions (such as division sites, cell poles, and hyphal tips) where AFP1 accumulates may be less compact and cross-linked, which allows AFP1 to approach the targets to engage in an interaction facilitated via mannose binding.

Although the percentages of protein sequence identity among the U. maydis CDAs are relatively low (11), AFP1 targets most UmCDAs probably via recognizing a conserved distorted (β/α) 8-barrel structure of NodB domain shared by the carbohydrate esterase family 4 (CE4) members (46). The findings of the AFP1-NodB interaction and AFP1* not interacting with the NodB domain and not binding mannose allow us to propose that the mannose binding of AFP1 functions in facilitating the interactions with mannosylated CDAs, which is further supported by the finding that AFP1 reduced binding to the actively growing sites if the mannosylation of targets is reduced, i.e., in Δ*pmt4*. Since C. graminicola GLRG_386 and GLRG_7915 proteins are the orthologs of UmCda4 and UmCda5 and interacted with AFP1, we expect that AFP1 binding at the cell poles of C. graminicola conidia where the germ tube and appressoria emerge could at least prevent fungal penetration and arrest their growth (47).

PMTs form a heterodimer or homodimer in Saccharomyces cerevisiae (48). Loss of *pmt4* significantly impacts the *O*-mannosylation of membrane proteins to which cell surface hydrophobicity and cell wall integrity defects may be attributed (49–52). U. maydis PMTs may form a dimer complex to mannosylate their UmCDA targets that bear a Ser-Thr stretch before the GPI anchor, which is missing in UmCda4. Residual mannosylation of UmCda1 and UmCda2 expressed in the U. maydis Δ*pmt4* background suggests that other U. maydis PMTs, such as Pmt1 and Pmt2, may also *O*-mannosylate UmCda1 and UmCda2 proteins. The status of Pmt4 mannosylation on the remaining four UmCDAs that localize to the bud tips/necks remains unknown. UmCda7 could be a potential candidate, as it also localizes to the cell division region, and the Δ*cda7* mutant exhibits reduced fungal virulence (11), a phenotype shared by the Δ*pmt4*. Due to the essential role of Pmt2 in U. maydis cell viability (38), it is impossible to delete all *PMT* genes to study the role of *O*-mannosylation in UmCDA localization and to understand the PMT-CDA specificity. AFP1 may have different affinities for UmCDAs based on various degrees of *O*-mannosylation and *N*-glycosylation on UmCDAs. Alternatively, a specific glycan pattern by a different combination of PMT dimers and other glucosidases may also contribute to or determine the interaction affinity. Our findings support a scenario in which the antifungal activity of mannose-binding AFP1 protein is coupled with at least the PMT-dependent *O*-mannosylation of UmCDAs or other glycoproteins.

AFP1 targets fungal cells by either recognizing the conserved NodB structure of CDAs or binds to specific glycan patterns of non-CDA glycoproteins to increase chitin levels and may alter cell wall plasticity to restrict fungal cell growth. These glycoproteins likely have related functional roles in regulating chitin biosynthesis and maintaining cell wall architecture. For example, loss of the S. cerevisiae GPI-anchored Gas1 associated with cell wall polymer assembly increases chitin levels and cell wall thickness and severely reduces growth rate and cell viability (53). This suggests that similar inhibitory effects can also be achieved via modulating the activity of other cell surface glycoproteins. A recent report showed that the DUF26 domains of rice OsRMC are required for both mannose binding and interacting with fungal CBM1 domain proteins, and AFP1 does not interact with CBM1 protein (54), which suggests the specificity of DUF26 domain-containing family protein.

The similar localization patterns of chitin synthases (CHS) and CDAs in the U. maydis sporidial cells (27, 55) may indicate an interaction of UmCDA with UmCHS to access *de novo* nascent chitin substrates and effectively convert them, as has been speculated for decades. If this hypothesis holds, the AFP1-UmCDA interactions might negatively impact the U. maydis CDA-CHS interactions, thereby blocking UmCDA accessibility to chitin and affecting the chitin conversion. AFP1-bound U. maydis cells had increased chitin levels in a zebra pattern, which was also shown in multiple *CDA* deletion strains (11). U. maydis multiple CDA deletion mutants and AFP-bound cells were viable at neutral pH, but they had severe growth defects at low pH, at which AFP1 displays antifungal activity. Previous studies report that the degree and pattern of chitin deacetylation and pH highly influence the antimicrobial activity of chitosan (56–58). We therefore speculate that instead of completely depleting CDA activity, AFP1 might modulate the degree and pattern of deacetylation in chitin, which could be toxic to AFP1-bound cells at a low pH. In addition to the inhibition of CDAs, the cumulative inhibition of other glycoproteins by AFP1 explains our observations of why the relatively moderate inhibition of CDA activity by AFP1 can have a dramatic effect on fungal viability.

In summary, our study on maize AFP1 provides molecular insights into understanding the antifungal mechanisms of plant CRRSP proteins. We demonstrate that the AFP1 targets conserved fungal CDAs and other glycoproteins and displays antagonistic effects against several fungi tested and suggest its high potential to be developed into an antifungal agent in controlling fungal diseases to reduce threats posed by the fungal kingdom.

## MATERIALS AND METHODS

### Strain construction and growth conditions.

The haploid solopathogenic Ustilago maydis SG200 strain was used as a reference strain in this study (22, 59). Strains used and generated in this study are listed in Table S1A in the supplemental material. Plasmid construction using either Gibson Assembly or standard cloning methods is described in Table S1B. Primers used in each generated plasmid are listed in Table S1C. A PCR-based approach was used to generate mutants (60, 61). For gene integration into the *ip* locus or *mig2-6* locus, plasmids containing a carboxin-resistant *ip* allele (*ip*R) (62) or an *mig2-6* allele were linearized with the restriction enzymes SspI or AgeI or EcoNI and subsequently inserted via homologous recombination. Transformation of U. maydis and genomic DNA isolation were performed as described. Positive U. maydis transformants were verified by Southern blotting.

10.1128/mbio.00093-23.9 TABLE S1 Strains, plasmids, and oligonucleotides used in this study. Download Table S1, PDF file, 0.3 MB. Copyright © 2023 Ma et al. 2023 Ma et al. https://creativecommons.org/licenses/by/4.0/ This content is distributed under the terms of the Creative Commons Attribution 4.0 International license.

U. maydis strains were grown at 28°C in a liquid YEPSL medium (0.4% yeast extract, 0.4% peptone, and 2% sucrose) and on potato dextrose agar (PDA; 2.4% potato dextrose broth and 2% agar). S. cerevisiae cells were grown on YPD agar (BD Difco, USA) at 28°C, and C. graminicola CgM2 was grown on oatmeal agar (BD Difco, USA) with continuous exposure to daylight at room temperature.

### Gene expression analysis.

SG200 cell pellets were collected after cells grown in liquid YESPL medium reached an optical density at 600 nm (OD_600_) of 0.6. C. graminicola (CgM2) conidial cells were harvested from oatmeal agar plates. S. cerevisiae cell pellets were harvested after the 4 h incubation in YPD liquid medium, 1× PBS (pH 7.2), and 10 mM MES (pH 5.5) with an initial OD of 0.4. The cell pellets were washed with sterilized water and frozen in liquid nitrogen. Total RNA extracted using TRIzol (Invitrogen) was subject to DNase treatment (Promega; catalog no. M6101) according to the manufacturer’s recommendation. The cDNA preparation and quantitative RT-PCR analysis were performed as described previously (8). The expressions of U. maydis peptidyl-prolyl isomerase (*PPI*) and S. cerevisiae *ACTIN* genes were used for normalization. To analyze CgM2 *CDA* gene expression using semiquantitative RT-PCR, *ACTIN* gene expression was used for normalization. PCR amplification was programmed as follows: 95°C for 25 s, 60°C for 25 s, and 72°C for 45 s. After 33 cycles for *CDA* amplification and 28 cycles for *ACTIN*, PCRs were further incubated at 72°C for 3 min and then chilled at 10°C. Gel images were acquired using the Molecular Imager Gel Doc XR+ system (Bio-Rad), and densitometric analysis was performed using ImageJ (63).

### Immunolocalization of AFP1 and CDA.

The purification of AFP1 proteins from Nicotiana benthamiana was performed as described (8). To visualize the localization of AFP1-His on sporidial cells, a final volume of 200 μL of 1× PBS buffer (pH 7.2) containing 4 μg AFP1-His proteins and OD of 1 of cells were incubated for 4 h at 28°C. To visualize AFP1-His localization on germinated spores of FB1 × FB2, spores were collected from infected maize leaves and allowed to germinate on potato dextrose (PD) agar containing 150 μg/mL ampicillin and 35 μg/mL chloramphenicol at 28°C as described (26). Germinated spores on PD agar were removed, washed, and incubated with 20 μg AFP1-His proteins in a final volume of 200 μL PBS for 4 h at 28°C. The cells/spores were washed with PBS before immunostaining using the primary anti-His antibody and Alexa Fluor 488 (AF488)-conjugated secondary antibody as described (8).

To visualize the localization of HA-tagged CDA proteins, sporidial cells were prewashed before being subjected to immunostaining using primary anti-HA antibody and AF488-conjugated secondary antibody (8) in 10 mM MES buffer (pH 5.5). To visualize the protein localization on filaments of U. maydis, cells were induced using hydroxyl fatty acid on parafilm as described (8), followed by either an initial incubation with AFP1-His proteins as described above or immunostaining. AF488 fluorescence was excited at 488 nm and subsequently detected at 500 to 540 nm using an Axio Observer fluorescence microscope equipped with Axiocam 702 monochrome camera (Zeiss, Germany). Images were processed using ZEN 3.2 imaging software (Zeiss).

### Colocalization and acceptor photobleaching.

The fluorescence-labeled AFP1-His proteins were prepared according to the manufacturer's instructions of the DyLight 550 antibody labeling kit (Thermo Fisher; catalog no. 84530). An OD_600_ of 1 of cells was washed with MES buffer before immunostaining using the primary anti-HA antibody and AF488-conjugated secondary antibody to localize CDA proteins or stained chitin with wheat germ agglutinin-conjugated Alexa Fluor 488 (WGA-AF488; Molecular Probes, Karlsruhe, Germany). The immunostained or WGA-AF488 stained cells were washed with MES buffer, followed by incubation with 4 μg of DyLight 550-labeled AFP1-His proteins (AFP1^550^) in a final volume of 200 μL at room temperature for 4 h. The AFP1^550^-treated cells were washed and visualized using epifluorescence microscopy.

Acceptor photobleaching measurements were performed using a confocal microscope (Zeiss LSM880). The donor (AF488) channel was excited at 488 nm and detected through a 500- to 540-nm emission filter. The acceptor (DyLight 550) channel was excited at 561 nm and detected through a 580- to 630-nm emission filter. The images in the donor and acceptor channels were acquired before and following acceptor fluorescent bleaching. Regions of interest (ROIs) that overlap the donor-acceptor fluorescence were selected for photodestruction by applying 100% laser power at a wavelength of 561 nm for 120 iterations. Evaluation of images was analyzed using the ZEN 3.0 software. The FRET efficiency was calculated as follows: *E*_FRET_ = [*I*_Donor(post)_ − *I*_Donor(pre)_]/*I*_Donor(post)_, where *I*_Donor(pre)_ and *I*_Donor(post)_ are the donor fluorescence intensities prior to and following photodestruction of the acceptor.

### Chitin staining.

SG200 cells were treated with or without 10 μg AFP1 in 10 mM MES (pH 5.5) overnight at room temperature, washed, and chitin stained with 1 μg/mL WGA-AF488 for 10 min before visualized by microscopy and compared under the same parameter setting. Images of WGA-stained cells were acquired, and intensities of stained cells were individually quantified and analyzed using the Trainable Weka Segmentation plugin in ImageJ (63).

### Live-cell time-lapse imaging.

Spores from FB1 × FB2-infected cobs of the maize variety Gaspe Flint were collected and germinated on PD agar as described (11). Germinated spores were harvested, incubated with 0.1 μg/μL AFP1-His^550^ proteins in 10 mM MES buffer for 4 h, and mixed with agar to a final concentration of 2%, which contained 2% PD medium, before spotting on a chambered coverslip (Ibidi; catalog no. 80827). Image series were acquired using the Olympus DeltaVision Core microscope system with a 63× water immersion objective. The frame time was set at 10 min, and a total of 42 frames were acquired. For monitoring AFP1 inhibition on sporidial cells, SG200 cells (OD_600_ = 0.002) were incubated with AFP1-His proteins in MES buffer for 4 h at room temperature before spotting on a chambered coverslip to acquire images. The frame time was set at 5 min, and a total of 85 frames were acquired in a 7-h duration.

### Pulldown assay.

Cells expressing secreted Strep-tagged CDA proteins were grown to an OD_600_ of 0.6 at 28°C. The 50-mL culture supernatant containing Strep-tagged CDA proteins was concentrated to 1 mL and exchanged to buffer W (100 mM Tris-HCl, 150 mM NaCl, 1 mM EDTA, pH 7.5) using 10-kDa cutoff centrifugal filters (Sartorius, Germany) before incubation with 25 μL of Strep-Tactin XT agarose beads (IBA Lifesciences, Germany) overnight at 4°C in the presence of protease inhibitor cocktail (Roche, Switzerland). The beads were washed with buffer W, incubated with 0.1 μg AFP1-His proteins in a final volume of 500 μL stringency buffer (100 mM Tris-HCl, 0.3 M NaCl, 0.1% NP-40, and 1× protease inhibitor cocktail, pH 7.5) at 4°C for 2 h, and then washed with the same buffer. The bound proteins were removed by boiling in sample buffer and subjected to immunoblotting using mouse monoclonal anti-6×His (Yao-Hong Biotech. Inc., Taiwan) and Strep-tag II (IBA Lifesciences, Germany) primary antibodies and the goat anti-mouse IgG-horseradish peroxidase (HRP)-conjugated secondary antibody (Thermo Scientific, USA). For the interaction of Cda4 and AFP1, concentrated culture supernatant containing Cda4HA was incubated with HA-agarose beads (Sigma; catalog no. A2029) in binding buffer (25 mM Tris-HCl, 0.3 M NaCl, and 0.1% NP-40, pH 7.5) at 4°C for 4 h. The Cda4-bound beads were washed with the same buffer, followed by incubation with 0.2 μg of AFP1-His proteins in a stringent buffer (25 mM Tris-HCl, 0.5 M NaCl, and 0.1% NP-40, pH 7.5) overnight at 4°C. The beads were washed with the same buffer and boiled in a sample buffer to remove the bound proteins for the immunoblotting analysis using mouse monoclonal anti-HA and anti-6×His antibodies (Yao-Hong Biotech, Inc.).

### CDA activity assay.

Cells expressing secreted CDA-Strep proteins were cultured in YEPSL liquid medium until the OD_600_ reached 0.6. A total of 50 mL culture supernatant was concentrated and exchanged with buffer W using 10-kDa cutoff centrifugal filters (Sartorius, Germany). Fifty microliters of Strep-Tactin XT agarose beads was added to the concentrated sample in a final volume of 1 mL containing 1× protease inhibitor cocktail and incubated overnight at 4°C. The beads were washed with the same buffer and aliquoted evenly into three tubes before 10 μg of AFP1-His, AFP1*-His, or bovine serum albumin (BSA) was added to a final volume of 350 μL buffer (50 mM NaHCO_3_, pH 7.0). The incubation was carried out at 4°C for 8 h before adding GlcNAc_5_ (A5; Megazyme; catalog no. O-CHI5) to a final concentration of 0.625 mg/mL, and we continued the incubation at 37°C for 16 h. The reaction mixture was centrifuged, and the release of acetate in the supernatant was measured by following the manufacturer’s instructions for the acetic acid test kit (R-Biopharm Inc.; catalog no. 10148261035).

### Lectin blot assay.

Immunoprecipitated CDA proteins from culture supernatant were separated on SDS-PAGE and transferred to a polyvinylidene difluoride (PVDF) membrane. The membrane was preincubated with radioimmunoprecipitation assay (RIPA) buffer (Sigma; catalog no. R0278) for 1 h before incubation with 0.01 μg/mL concanavalin A (Sigma; catalog no. C5275) overnight at 4°C. The membrane was washed and subjected to immunoblotting analysis using anti-lectin antibody (Sigma; catalog no. C7401).

### Deglycosylation assay.

Total protein extracted from cell pellet was incubated with deglycosylation enzyme mix (NEB; catalog no. P6039) or PNGase F (NEB; catalog no. P0704) in 20 μL of supplied buffer according to the manufacturer´s protocol.

### Yeast two-hybrid assay.

Yeast two-hybrid assays were performed as described (64). Yeast (AH109) transformants containing the desired plasmids were screened on a selective dropout (SD) medium lacking tryptophan (W) and leucine (L) (Clontech). Protein interactions were assessed on SD selection medium lacking SD-Leu-Trp (LW), histidine (H), and adenine (A) or lacking LWH and containing 3-amino-1,2,4-triazole (3-AT) after 3 to 5 days incubation at 28°C. To detect the expression of proteins in yeast transformants, an OD_600_ of 1 of yeast cells was lysed in a buffer containing 1% β-mercaptoethanol and 0.25 M NaOH. The supernatant fraction was trichloroacetic acid (TCA) precipitated and then centrifuged. The protein pellet was dissolved in HU buffer (100 mM Tris-HCl pH 6.8, 8 M urea, 5% SDS, 0.1 mM EDTA, and 0.1% bromophenol blue) and analyzed by immunoblotting.

### Growth inhibition assay.

U. maydis or S. cerevisiae BY4742 cells (OD_600_ = 0.001) were incubated with 20 μg AFP1 proteins in a 200 μL of 10 mM MES buffer (pH 5.5) at 28°C for 4 h. The cells were serial diluted, spread on YPD agar plates (three plates per each dilution), and incubated at 28°C for 2 days until colonies appeared. Colony numbers were then manually counted.
