# Supplementary material for: Maize Antifungal Protein AFP1 Elevates Fungal Chitin Levels by Targeting Chitin Deacetylases and Other Glycoproteins
Source: mBio. 2023 Mar 22;14(2):e00093-23. doi: 10.1128/mbio.00093-23 (PMC10128019; doi:10.1128/mbio.00093-23)
Supplement: FIG S1 [file mbio.00093-23-s0001.pdf]

FIG S1

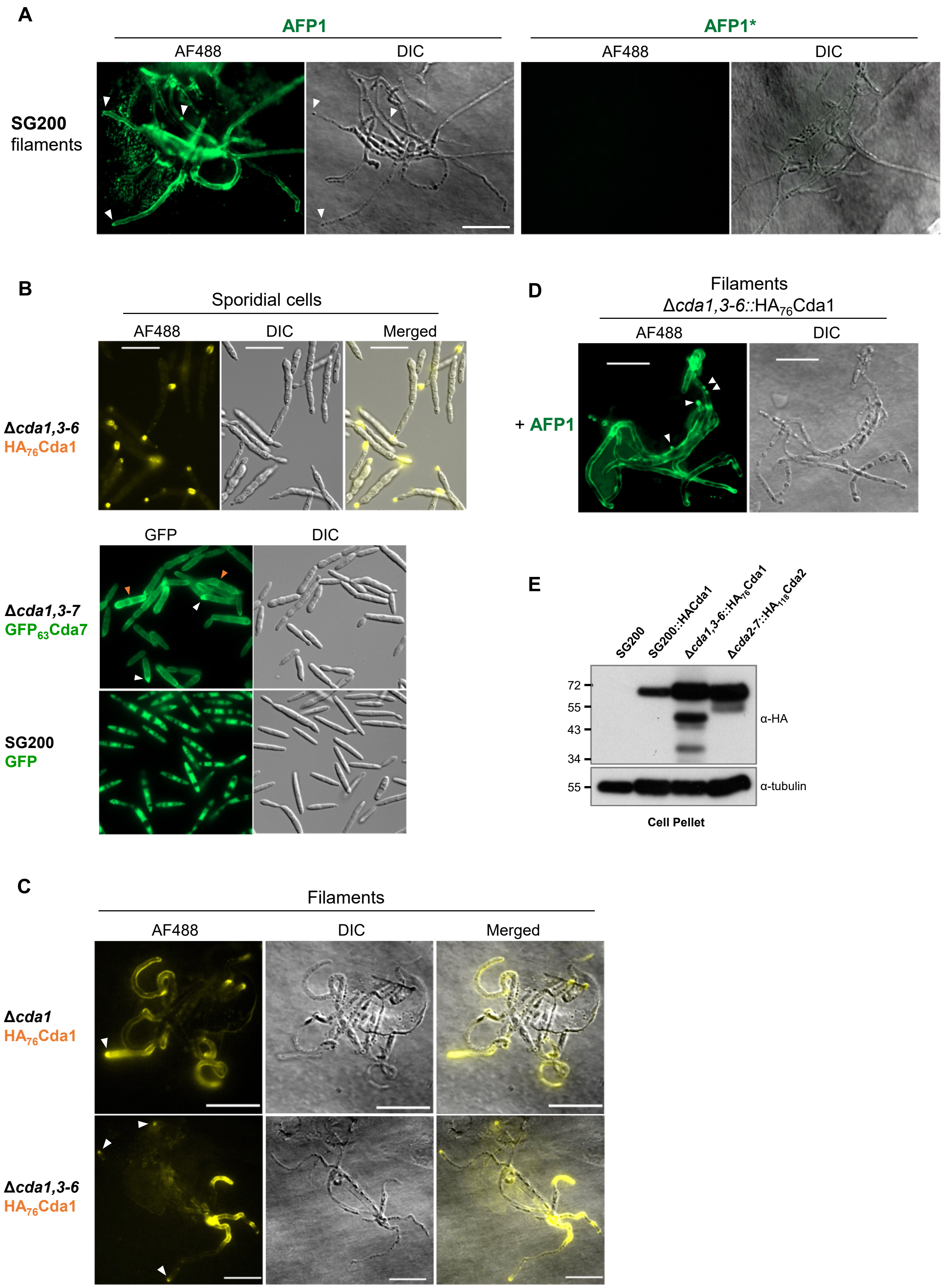
A
AFP1
AFP1*
AF488
DIC
AF488
DIC
SG200
filaments
B
Sporidial cells
AF488
DIC
Merged
Δcda1,3-6
HA$_{76}$Cda1
GFP
DIC
Δcda1,3-7
GFP$_{63}$Cda7
SG200
GFP
C
Filaments
AF488
DIC
Merged
Δcda1
HA$_{76}$Cda1
Δcda1,3-6
HA$_{76}$Cda1
D
Filaments
Δcda1,3-6::HA$_{76}$Cda1
AF488
DIC
+ AFP1
E
SG200
SG200::HACda1
Δcda1,3-6::HA$_{76}$Cda1
Δcda2-7::HA$_{118}$Cda2
72
55
43
34
55
α-HA
α-tubulin
Cell Pellet

FIG S1 Immunolocalization of AFP1 and CDAs in *U. maydis* cells. Bars, 20 μm.

(A, C-D) Filaments of indicated strains were induced by hydroxyl fatty acids on the hydrophobic surface, followed by immunostaining using an anti-His antibody or anti-HA antibody and an AF488-conjugated secondary antibody to localize AFP1 or HA_{76}Cda1. Arrowheads indicate AFP1 or HA_{76}Cda1 fluorescence on hyphal tips. (B) Localization of HA_{76}Cda1 and GFP_{63}Cda7 in sporidial cells of indicated *CDA* multiple mutants which constitutively expressed CDA proteins under constitutive promoter *otef.* Non-secreted cytosolic GFP expressed in SG200 was shown. White and orange arrowheads indicate the tip and septa localization of GFP_{63}Cda7, respectively. (E) Immunoblot analysis of HA-tagged CDA protein expression. HA-tagged CDA proteins were constitutively expressed in indicated strains, which grown in YEPSL liquid medium to OD_{600} of 0.6-0.7 before harvested. Total proteins from cell pellets were prepared, and subjected to immunoblot analysis using anti-HA or anti-tubulin antibodies as indicated.
