# Supplementary material for: Maize Antifungal Protein AFP1 Elevates Fungal Chitin Levels by Targeting Chitin Deacetylases and Other Glycoproteins
Source: mBio. 2023 Mar 22;14(2):e00093-23. doi: 10.1128/mbio.00093-23 (PMC10128019; doi:10.1128/mbio.00093-23)
Supplement: FIG S2 [file mbio.00093-23-s0002.pdf]

FIG S2

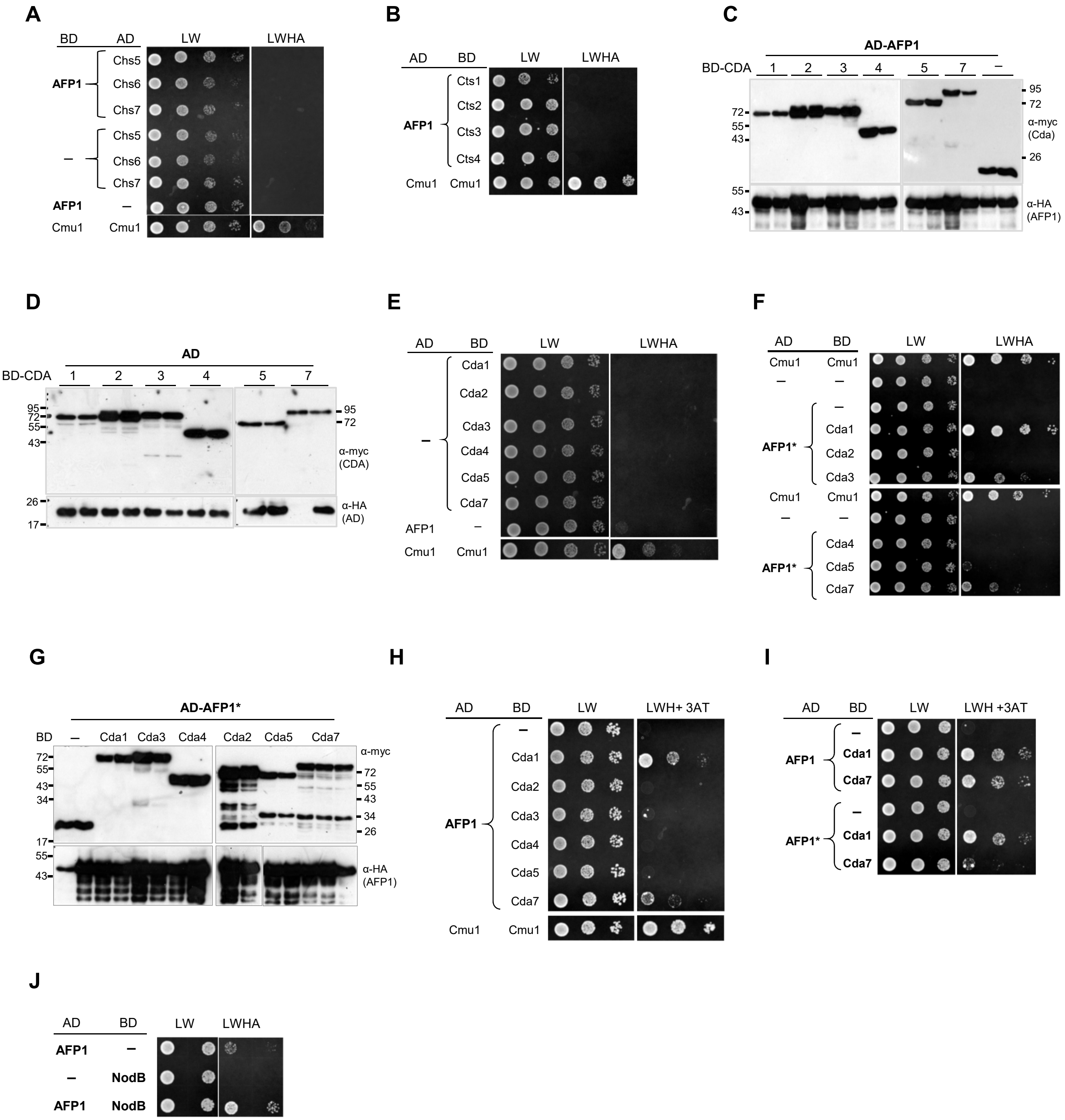
A
BD AD LW LWHA
AFP1 Chs5 Chs6 Chs7
– Chs5 Chs6 Chs7
AFP1 –
Cmu1 Cmu1
B
AD BD LW LWHA
AFP1 Cts1 Cts2 Cts3 Cts4
Cmu1 Cmu1
C
AD-AFP1
BD-CDA 1 2 3 4 5 7 –
95 72 55 43 26
α-myc (Cda)
α-HA (AFP1)
D
AD
BD-CDA 1 2 3 4 5 7
95 72 55 43 26 17
α-myc (CDA)
α-HA (AD)
E
AD BD LW LWHA
– Cda1 Cda2 Cda3 Cda4 Cda5 Cda7
AFP1 –
Cmu1 Cmu1
F
AD BD LW LWHA
Cmu1 Cmu1
– –
AFP1* – Cda1 Cda2 Cda3
Cmu1 Cmu1
– –
AFP1* Cda4 Cda5 Cda7
G
AD-AFP1*
BD – Cda1 Cda3 Cda4 Cda2 Cda5 Cda7
72 55 43 34 26 17
α-myc
α-HA (AFP1)
H
AD BD LW LWH+ 3AT
AFP1 – Cda1 Cda2 Cda3 Cda4 Cda5 Cda7
Cmu1 Cmu1
I
AD BD LW LWH +3AT
AFP1 – Cda1 Cda7
AFP1* – Cda1 Cda7
J
AD BD LW LWHA
AFP1 –
– NodB
AFP1 NodB

FIG S2 Yeast two-hybrid assays for the interactions of AFP1 with UmCHSs (chitin synthases), UmCTSs (chitinases), and UmCDAs (chitin deacetylases).

Yeast transformants containing two plasmids expressing indicated proteins fused to GAL4 activation domain (AD) or binding domain (BD) without signal peptide were grown on SD-Leu/-Trp (LW), SD-Leu/-Trp/-His/-Ade (LWHA), or SD-LWH containing 1mM of 3AT (3-amino-1,2,4-triazole) plates for 2-3 days. –, empty vector; Chorismate mutase (Cmu1) served as the positive control; AD/BD-AFP1, AD/BD-CDA, and AD-CHS/BD served as the negative controls. Similar results were observed in at least two independent experiments. Chs5 (UMAG_10277); Chs6 (UMAG_10367); Chs7 (UMAG_05480); Cts1 (UMAG_10419); Cts2 (UMAG_02758); Cts3 (UMAG_06190); Cts4 (UMAG00695). NodB domain of Cda1 consists of 198 amino acids from 108 to 305. (C-D, G) Immunoblot analysis of indicated AD- and BD-fusion protein expression in yeast transformants. Yeast transformants were grown in YPD liquid medium until OD_{600} reached 0.7-0.8. One OD of cell pellet was collected, lysed, and TCA precipitated and analyzed by immunoblots using indicated antibodies. –, empty vector; Two independent clones were selected for immunoblotting analysis. Only clones expressing the expected proteins were used in Y2H analysis.
