# Supplementary material for: Maize Antifungal Protein AFP1 Elevates Fungal Chitin Levels by Targeting Chitin Deacetylases and Other Glycoproteins
Source: mBio. 2023 Mar 22;14(2):e00093-23. doi: 10.1128/mbio.00093-23 (PMC10128019; doi:10.1128/mbio.00093-23)
Supplement: FIG S3 [file mbio.00093-23-s0003.pdf]

A

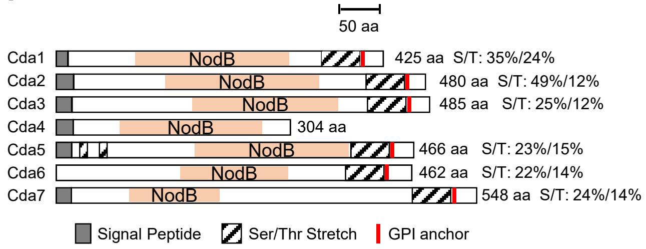

B

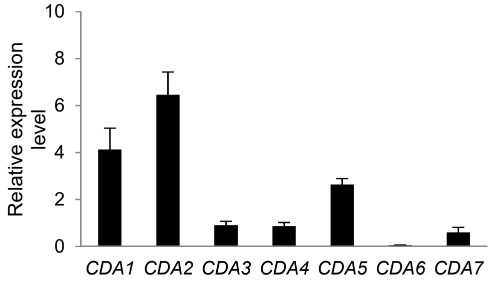

C

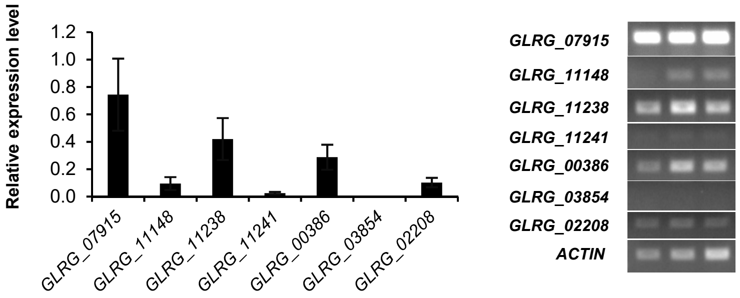

D

	Cda1	Cda2	Cda3	Cda4	Cda5	Cda6	Cda7	Predicted by OMA
**GLRG_7915**	24.7	26.4	25.9	23.5	28.1	24.4	26.4	Cda5
**GLRG_11148**	26.0	25.6	25.7	21.5	24.9	25.1	25.0	/
**GLRG_11238**	26.5	28.7	27.2	23.5	26.4	25.9	25.4	Cda2
**GLRG_11241**	30.3	28.1	26.9	25.7	28.3	26.9	25.5	/
**GLRG_00386**	23.9	21.7	21.8	29.5	24.5	23.1	21.2	Cda4
**GLRG_3854**	22.5	22.2	23.7	27.6	23.7	23.4	20.7	/
**GLRG_2208**	24.3	26.2	23.5	18.8	22.3	23.2	23.9	/

FIG S3 *CDA* gene expression in *U. maydis* and *C. graminicola*.

(A) Schematic drawings of *U. maydis* CDA proteins with indicated signal peptides, CDA domains, predicted GPI anchors, and Serine/Threonine (S/T) stretch region. The percentage of S/T enriched in the last 50 amino acid sequences before the GPI anchor is shown. a. a., amino acids. Cda1 (UMAG_00638); Cda2 (UMAG_01143); Cda3 (UMAG_11922); Cda4 (UMAG_01788); Cda5 (UMAG_02019); Cda6 (UMAG_05792); Cda7 (UMAG_02381). (B) Total RNA was extracted from SG200 cells grown in a YEPSL liquid medium and subjected to quantitative RT-PCR. Expression levels of *the U. maydis CDA* gene were normalized relative to the constitutively expressed *Peptidyl-Prolyl Isomerase* (*PPI*). Values represent the mean ± sd of the three biological replicates. (C) Total RNA extracted from *C. graminicola* conidial cells grown on agar plates was subjected to semi-quantitative RT-PCR. Values were obtained by quantified intensities of PCR bands in the right panel using ImageJ software and were normalized relative to the constitutively expressed *ACTIN* gene. Data represent the mean ± sd of the three biological replicates. (D) The percentages of amino acid sequence identity between CDAs of *U. maydis* and *C. graminicola* determined by pairwise sequence alignment calculated using EMBOSS Stretcher (https://www.ebi.ac.uk/Tools/psa/emboss_stretcher/). The orthologs of UmCDAs in CgM2 are predicted by using the server OMA (Orthologous Matrix) (https://omabrowser.org/oma/home/) and highlighted in red.
