# Supplementary material for: Maize Antifungal Protein AFP1 Elevates Fungal Chitin Levels by Targeting Chitin Deacetylases and Other Glycoproteins
Source: mBio. 2023 Mar 22;14(2):e00093-23. doi: 10.1128/mbio.00093-23 (PMC10128019; doi:10.1128/mbio.00093-23)
Supplement: FIG S4 [file mbio.00093-23-s0004.pdf]

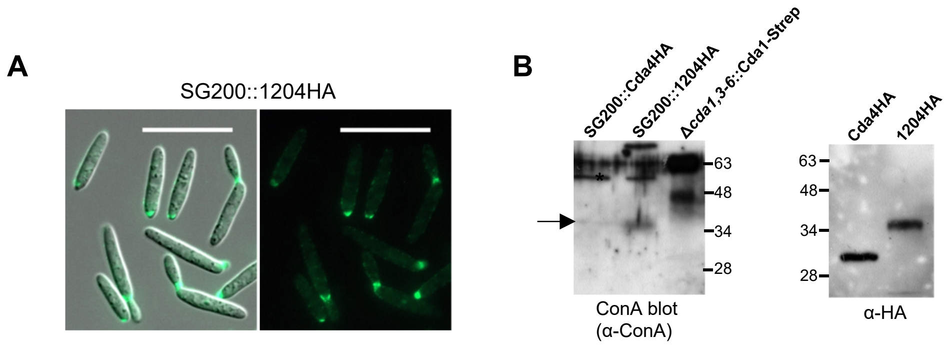

FIG S4 Localization and ConA blot analysis of UMAG1204

(A) Immunolocalization of non-CDA mannoprotein UMAG1204HA expressing in SG200 cells under constitute promoter *otef*. Bar, 20 µm. (B) Mannosylation of UMAG1204 by ConA blot analysis. Tagged proteins were constitutively expressed in indicated strains and immunoprecipitated from culture supernatants, separated on SDS-PAGE, incubated with and without ConA (Concanavalin A), and blotted against anti-ConA and anti-HA antibodies. Arrow indicates 1204HA proteins.
