# Supplementary material for: Maize Antifungal Protein AFP1 Elevates Fungal Chitin Levels by Targeting Chitin Deacetylases and Other Glycoproteins
Source: mBio. 2023 Mar 22;14(2):e00093-23. doi: 10.1128/mbio.00093-23 (PMC10128019; doi:10.1128/mbio.00093-23)
Supplement: FIG S5 [file mbio.00093-23-s0005.pdf]

FIG S5A Putative *O*-mannosylation sites in ScCDAs, CgCDAs, and UMAG1204

ScCda1

Site	Score
39	0.8686
52	0.7984

ScCda2

Site	Score
47	0.8334
49	0.7864

GLRG_386

Site	Score
30	0.8041

UMAG1204

Site	Score
46	0.824723
48	0.798481
52	0.939939
64	0.952892
69	0.909009
79	0.949121
81	0.984408
85	0.982707
86	0.988197
87	0.997816
91	0.986398
93	0.985646
100	0.9695
106	0.948973
107	0.986013
109	0.933134
111	0.971963
113	0.966761
114	0.979468
115	0.983874
117	0.969792
118	0.979012
121	0.984949
123	0.939675
124	0.861189

GLRG_7915

Site	Score
40	0.864599
41	0.851865
60	0.777174
92	0.725725
108	0.919859
115	0.789693
119	0.747508
121	0.760376
125	0.85838
128	0.737354
132	0.883051
134	0.847879
137	0.71706
142	0.841118
149	0.918821
156	0.847562
165	0.781644
166	0.814768
174	0.958453
175	0.790585
178	0.951268
179	0.943853
184	0.838865
186	0.82913
187	0.895824
195	0.833218
202	0.920492
203	0.918906
204	0.863696
208	0.938139
209	0.933369
212	0.850764
214	0.894548

GLRG_7915

Site	Score
215	0.906296
217	0.905072
218	0.902717
219	0.958095
220	0.944669
223	0.950683
224	0.938174
226	0.820602
230	0.790741
445	0.737492
448	0.889069
449	0.975711
450	0.922243
452	0.824637
454	0.93204
456	0.945329
459	0.951803
461	0.955653
463	0.928994
464	0.972097
465	0.958836
467	0.947743
469	0.932455
524	0.943944
527	0.906845
529	0.946542
530	0.987206
531	0.987999
532	0.975623
537	0.978149
539	0.939041
543	0.91008
547	0.730221

FIG S5B Putative *O*-mannosylation sites in *U. maydis* UmCDAs

UmCda1

Site	Score
29	0.844834
30	0.920223
33	0.90299
75	0.705468
334	0.861575
346	0.740475
347	0.893203
348	0.876396
349	0.878543
350	0.930404
354	0.948597
355	0.975606
356	0.967651
357	0.956971
359	0.96976
360	0.986395
361	0.971125
362	0.955192
363	0.991907
364	0.973984
366	0.954238
367	0.989608
368	0.969677
370	0.982256
371	0.987463
372	0.930864
374	0.972129
376	0.982835
378	0.959285
380	0.979975
383	0.957567
384	0.95938
386	0.893182
387	0.959799
389	0.962033
399	0.740633

UmCda2

Site	Score
34	0.898533
47	0.892403
53	0.954808
58	0.811638
59	0.940261
60	0.942317
61	0.966597
78	0.800474
79	0.832493
103	0.863595
105	0.937148
109	0.949325
112	0.965555
118	0.880091
125	0.76462
284	0.712868
286	0.84909
287	0.789719
368	0.814501
372	0.825635
381	0.899504
387	0.897451
394	0.833271
409	0.870305
410	0.862531
411	0.899515
413	0.939882
417	0.884772
419	0.908351
422	0.966649
423	0.986682
426	0.979076
429	0.940466
431	0.790494
432	0.967123
433	0.889202
434	0.928956
435	0.969516
437	0.978168
438	0.955478
440	0.958064
441	0.966452
442	0.813588
443	0.922895
444	0.962144
445	0.926957
447	0.92842
448	0.826967
449	0.939741
451	0.867605
453	0.845095
454	0.803287
455	0.762646

UmCda3

Site	Score
54	0.738953
58	0.911182
65	0.94428
66	0.893564
97	0.804032
126	0.758908
140	0.851414
400	0.742168
406	0.748576
422	0.963756
424	0.877395
426	0.849416
428	0.756568
430	0.938789
437	0.957581
438	0.966066
439	0.922723
440	0.938988
441	0.939692
445	0.975279
446	0.981567
449	0.938515
450	0.922666
451	0.846187
454	0.816686
457	0.848443

UmCda4

Site	Score
26	0.805412
29	0.830826
30	0.823892
36	0.821241
65	0.784744
290	0.881067

UmCda5

Site	Score
30	0.825097
31	0.875512
33	0.746087
35	0.911241
38	0.708039
39	0.937329
40	0.855086
50	0.717684
51	0.797858
60	0.801148
69	0.961828
70	0.852386
75	0.969438
76	0.953751
77	0.915296
78	0.882734
79	0.931536
80	0.933712
83	0.950834
84	0.969943
95	0.93172
105	0.706443
109	0.879265
113	0.848256
120	0.817369
122	0.8105
135	0.736763
141	0.881107
142	0.840373
143	0.855636
146	0.874708
148	0.815796
151	0.846267
400	0.770727
402	0.806037
403	0.796179
405	0.723691
410	0.893716
413	0.909808
415	0.803879
419	0.936174
420	0.976682
421	0.962169
427	0.935298
430	0.891227
431	0.979791
432	0.835181
437	0.804446
438	0.875162

UmCda6

Site	Score
37	0.758702
42	0.742959
110	0.71821
114	0.897218
363	0.769631
369	0.860155
373	0.714843
374	0.700218
375	0.851179
379	0.783086
404	0.932238
408	0.941058
412	0.96838
414	0.931194
415	0.983412
416	0.901216
422	0.938725
423	0.839741
425	0.838426
429	0.91457
432	0.700052
433	0.765638

UmCda7

Site	Score
37	0.815086
53	0.900691
55	0.868671
61	0.855819
82	0.721142
385	0.768368
432	0.799767
488	0.839192
492	0.970166
493	0.729549
495	0.886774
498	0.939289
500	0.911059
504	0.900491
505	0.906624
507	0.851062
509	0.789947
510	0.84382

FIG S5C Putative *O*-mannosylation sites in *U. maydis* UmCHS and UmCTS

UmChs5

Site	Score	Site	Score
6	0.84329	1328	0.80426
17	0.84036	1358	0.75012
22	0.97024	1360	0.70016
36	0.9081	1361	0.86485
37	0.87039	1373	0.83747
46	0.87924	1375	0.82661
78	0.92328	1377	0.90808
84	0.9824	1379	0.90844
92	0.70811	1399	0.84845
97	0.83402	1405	0.97478
104	0.85957	1408	0.97691
105	0.91075	1410	0.95471
106	0.91237	1412	0.97138
107	0.91383	1421	0.98341
110	0.85142	1422	0.99594
117	0.8582	1430	0.93484
129	0.89083	1437	0.91423
132	0.94747	1439	0.91367
135	0.92557	1442	0.92361
138	0.90239	1444	0.87457
145	0.88722	1447	0.93856
151	0.82812	1453	0.94797
153	0.85885	1459	0.94278
161	0.8292	1466	0.91833
174	0.70964	1479	0.96539
180	0.91401	1480	0.97032
192	0.83535	1481	0.94608
220	0.86125	1482	0.94905
221	0.75876	1483	0.97262
253	0.84323	1504	0.97275
256	0.94105	1505	0.96217
274	0.85571	1511	0.95746
295	0.95959	1513	0.96894
301	0.98102	1515	0.97253
303	0.93802	1523	0.9612
308	0.96684	1528	0.91455
318	0.86406	1531	0.96477
321	0.77313	1533	0.97511
495	0.75195	1535	0.91725
1286	0.73094	1546	0.96506
1304	0.84011	1550	0.94954
1306	0.78342	1554	0.89152
1308	0.82414	1558	0.92567
1311	0.89718	1567	0.9155
1312	0.77365	1581	0.97281
1319	0.95642	1586	0.97735
1321	0.88292	1589	0.98597
1327	0.8572	1596	0.85259

UmChs7

Site	Score
13	0.79145
25	0.85687
30	0.95215
40	0.96502
47	0.95907
49	0.87144
54	0.95391
64	0.90641
65	0.92743
71	0.80243
74	0.83305
204	0.71406
206	0.74558
215	0.84011
216	0.85724
310	0.81364
440	0.7079
928	0.83422
936	0.90567
938	0.75522
953	0.82192
1073	0.84416
1076	0.84308
1090	0.81764
1098	0.85819
1105	0.80898
1120	0.76942
1138	0.86487
1146	0.76682
1152	0.87587
1154	0.83656
1160	0.91392
1168	0.80484
1171	0.86145
1183	0.82657
1184	0.91176
1187	0.97662
1194	0.96627
1195	0.96418
1197	0.92233
1203	0.92835
1221	0.97097
1233	0.97043
1248	0.90457
1251	0.88858
1259	0.95594
1262	0.86016

UmChs6

Site	Score
2	0.72057
3	0.74529
12	0.93724
16	0.78507
18	0.91804
25	0.87082

UmCts1

Site	Score
23	0.87374
24	0.80909
26	0.79765
27	0.89162
31	0.88101
33	0.9093
37	0.92606
39	0.96811
43	0.95659
47	0.96465
49	0.9813
51	0.91279
56	0.94475
57	0.95065
59	0.95025
60	0.84086
61	0.93981
66	0.81092
70	0.83539
73	0.87906
74	0.96324
75	0.78591
76	0.97392
79	0.98469
80	0.98614
81	0.95519
83	0.96885
84	0.94078
87	0.94903
89	0.94977
90	0.93583
93	0.85694
108	0.70202

UmCts3

Site	Score
60	0.80961
63	0.86607
64	0.85392
65	0.71626
66	0.85723
67	0.75632
85	0.86231
98	0.84747
108	0.92195
112	0.84824
114	0.9337
115	0.92258
118	0.88689
121	0.94933
124	0.93648
129	0.94897
130	0.92206
132	0.92964
134	0.88693
138	0.97433
140	0.94184
141	0.92076
142	0.95888
144	0.98451
145	0.93737
146	0.9724
148	0.9872
152	0.97163
154	0.92497
156	0.88501
163	0.79695
415	0.83747
417	0.95555
419	0.96885
421	0.9305
422	0.97964
423	0.96861
424	0.93824
433	0.70942
434	0.80469

UmCts4

Site	Score
68	0.78378
405	0.7613
417	0.76607

FIG S5D Putative *N*-glycosylation sites in CDAs, UmCHS, UmCTS, and UMAG1204

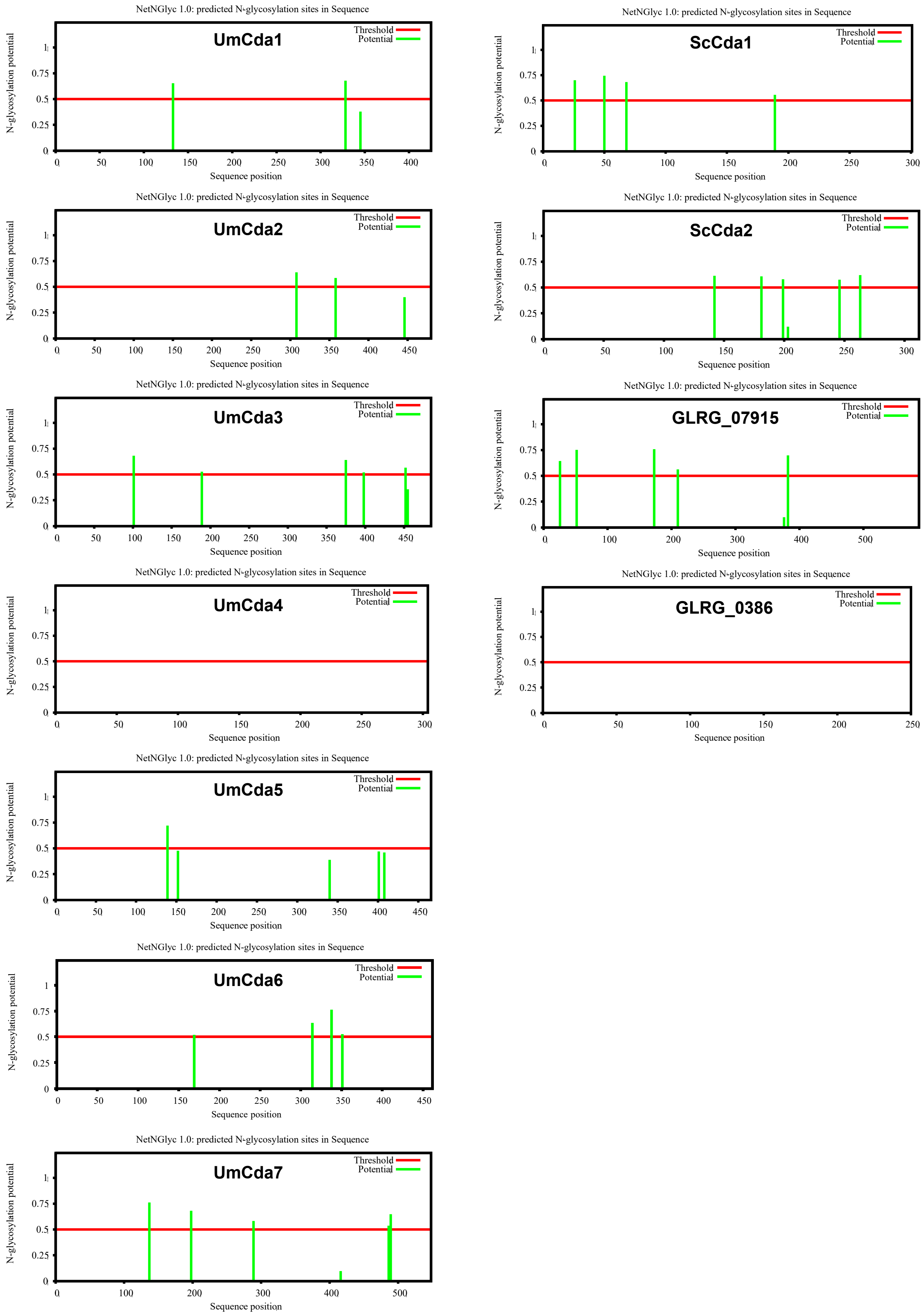

FIG S5D Putative *N*-glycosylation sites in CDAs, UmCHS, UmCTS, and UMAG1204

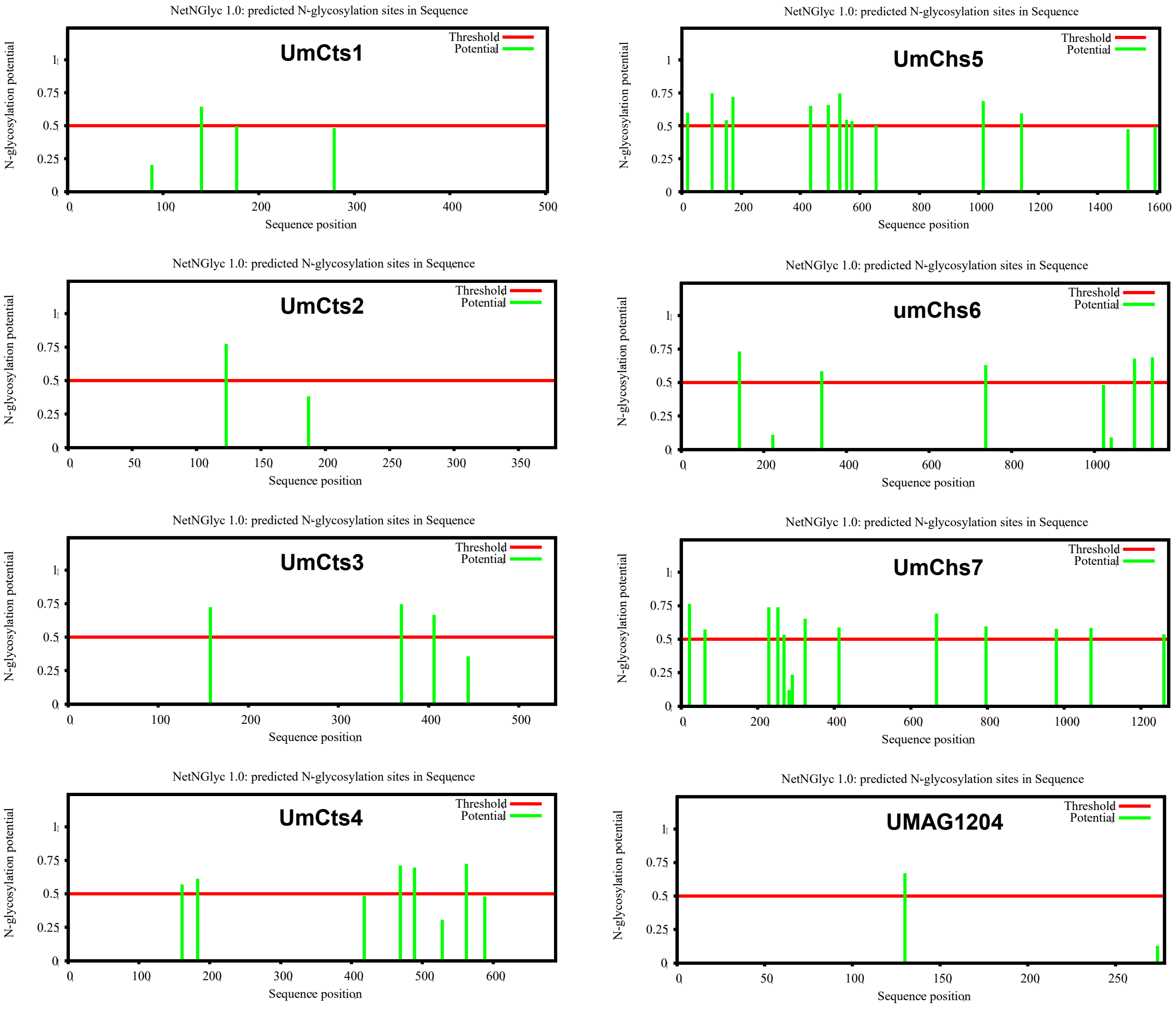

FIG S5 Prediction *O*- and *N*-glycosylation sites in UmCHSs, UmCTSs, CDAs and UMAG1204.

(A-C) *O*-mannosylation sites were predicted by the online NetOGlyc server (https://services.healthtech.dtu.dk/service.php?NetOGlyc-4.0). The cutoff value was set at ≥ 0.7. (A) Putative *O*-mannosylation sites for ScCDAs, CgCDAs, and UMAG1204. (B) Putative *O*-mannosylation sites for *U. maydis* UmCDAs. (C) Putative *O*-mannnosylation sites for *U. maydis* UmCHSs and UmCTSs. No mannosylation site was predicted for UmCts2. (D) Putative *N*-glycosylation sites of CDAs, UmCHSs, UmCTSs, and UMAG1204 proteins predicted by the online NetNGlyc server (https://services.healthtech.dtu.dk/service.php?NetNGlyc-1.0). No *N*-glycosylation site was predicted for UmCda4 and *C. graminicola* GLRC_0386.
