# Supplementary material for: Maize Antifungal Protein AFP1 Elevates Fungal Chitin Levels by Targeting Chitin Deacetylases and Other Glycoproteins
Source: mBio. 2023 Mar 22;14(2):e00093-23. doi: 10.1128/mbio.00093-23 (PMC10128019; doi:10.1128/mbio.00093-23)
Supplement: FIG S6 [file mbio.00093-23-s0006.pdf]

# FIG S6

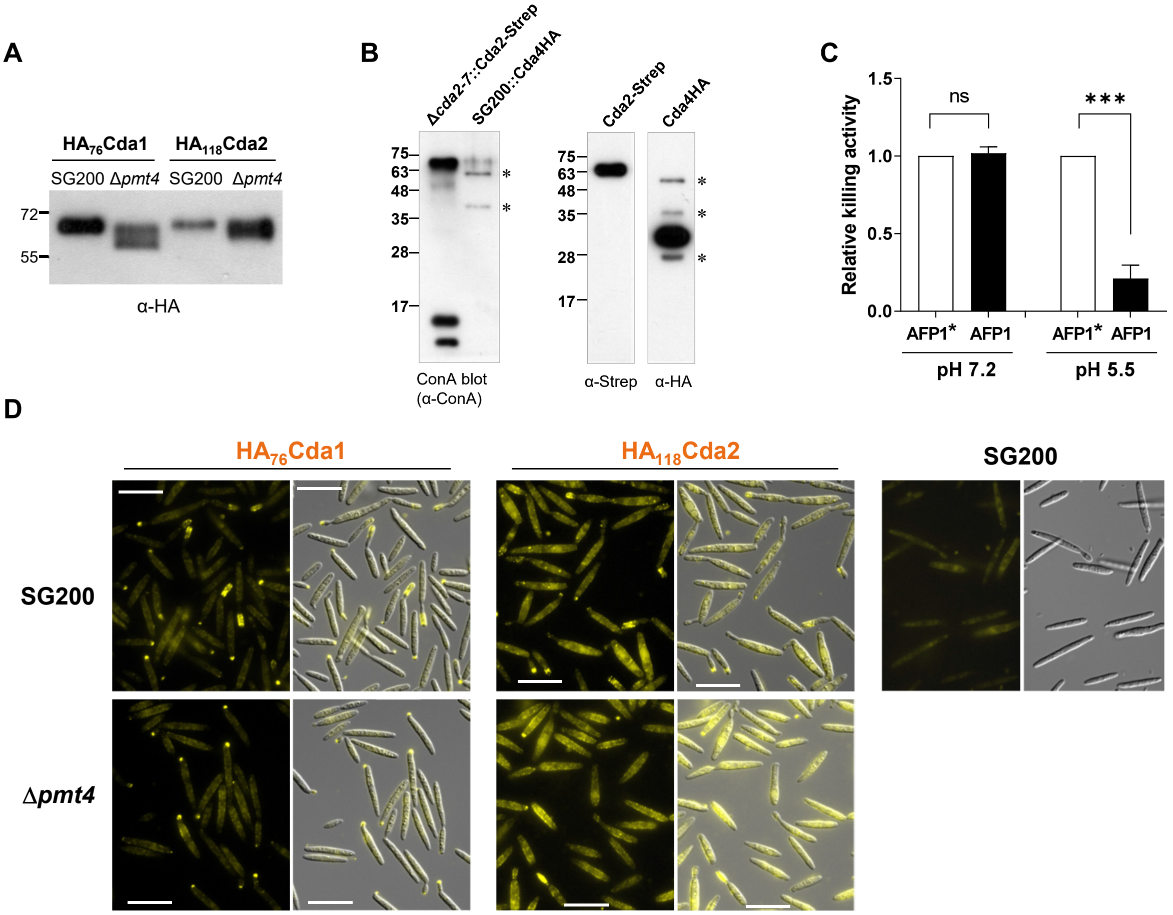
A
HA76Cda1
HA118Cda2
SG200 Δpmt4 SG200 Δpmt4
72
55
α-HA
B
Δcda2-7::Cda2-Strep
SG200::Cda4HA
Cda2-Strep
Cda4HA
75
63
48
35
28
17
ConA blot
(α-ConA)
α-Strep
α-HA
C
Relative killing activity
1.5
1.0
0.5
0.0
ns
***
AFP1* AFP1 AFP1* AFP1
pH 7.2
pH 5.5
D
HA76Cda1
HA118Cda2
SG200
SG200
Δpmt4

**FIG S6** Analysis of UmCDA mannosylation and localization and pH-dependent killing activity of AFP1.

(A) Total proteins from the cell pellet of indicated strains expressing respective CDA proteins under constitutive promoter *otef* were collected and separated on SDS-PAGE. The migration patterns of CDA proteins were analyzed by immunoblotting using an anti-HA antibody. (B) ConA recognized mannan of Cda2. Cda2-Strep and Cda4HA were constitutively expressed in indicated strains, immunoprecipitated from the culture supernatants, and analyzed by blotting with ConA and by immunoblotting using anti-ConA, anti-HA, and anti-Strep antibodies. *, non-specific protein bands. (C) The effect of pH on AFP1 killing activity. SG200 cells were incubated with AFP1/AFP1* in either PBS buffer (pH7.2) or MES buffer (pH 5.5) for 4 hours. The number of cells that survived in the AFP1 treatment was relative to the number of AFP1*-treated surviving cells, which was set as 1. Values represent the mean ± sd of three independent experiments. Asterisks indicate significant differences between two treatments determined by a two-tailed Student's *t*-test. *** $P$ values < 0.001. (D) Immunolocalization of $HA_{76}$Cda and $HA_{118}$Cda2. Cells expressing HA-tagged CDA proteins under constitutive promoter *otef* in indicated strains were subjected to immunostaining using an anti-HA antibody and an AF488-conjugated secondary antibody to localize CDA proteins. Bars, 20 μm. Right, SG200 cells subjected to immunostaining using an anti-HA antibody and AF488-conjugated secondary antibody served as negative control.
