# Supplementary material for: Maize Antifungal Protein AFP1 Elevates Fungal Chitin Levels by Targeting Chitin Deacetylases and Other Glycoproteins
Source: mBio. 2023 Mar 22;14(2):e00093-23. doi: 10.1128/mbio.00093-23 (PMC10128019; doi:10.1128/mbio.00093-23)
Supplement: FIG S7 [file mbio.00093-23-s0007.pdf]

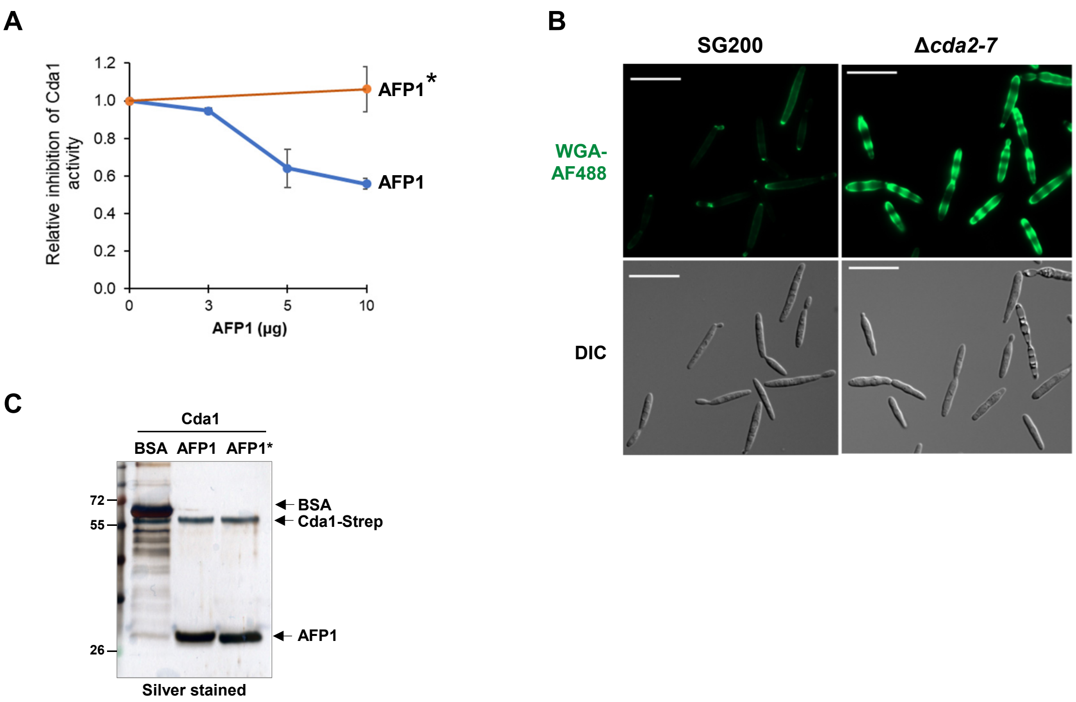

FIG S7 Inhibition of UmCda1 activity by AFP1 and chitin-staining analysis.

(A) UmCda1-Strep proteins bound on beads were pre-incubated with different amounts of AFP1-His, ranging from 0 to 10 µg (1 µM) AFP1 or 10 µg of AFP1*-His for 4 hours at 25 °C before the incubation with GlcNAc$_5$ for additional 2 hours. The hydrolytic activity towards GlcNAc$_5$ was determined. Data are means ± sd of three independent determinations. UmCda1 activities were relative to the activity at 0 µg of AFP1 (1 µM of BSA), which was set as 1. (B) Cells of indicated strains in the exponential phase were stained with WGA-AF488 to detect chitin. Bars, 20 µm. (C) Representative silver-staining gel image of indicated proteins loading in each reaction for CDA activity shown in Fig. 6A.
