# Supplementary material for: Maize Antifungal Protein AFP1 Elevates Fungal Chitin Levels by Targeting Chitin Deacetylases and Other Glycoproteins
Source: mBio. 2023 Mar 22;14(2):e00093-23. doi: 10.1128/mbio.00093-23 (PMC10128019; doi:10.1128/mbio.00093-23)
Supplement: FIG S8 [file mbio.00093-23-s0008.pdf]

FIG S8

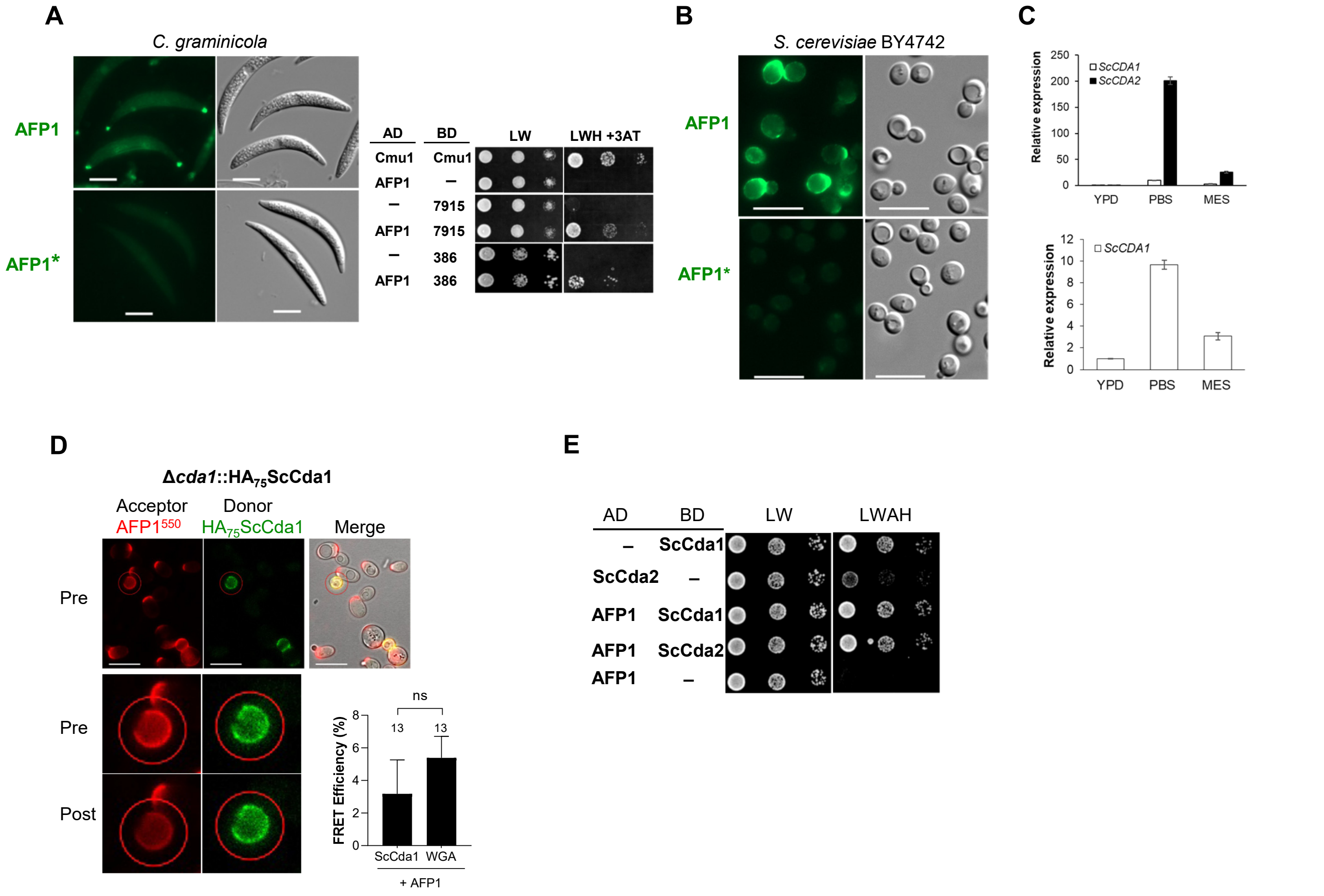
A
C. graminicola
AFP1
AFP1*
AD
BD
LW
LWH +3AT
Cmu1
Cmu1
AFP1
–
–
7915
AFP1
7915
–
386
AFP1
386
B
S. cerevisiae BY4742
AFP1
AFP1*
C
Relative expression
ScCDA1
ScCDA2
250
200
150
100
50
0
YPD
PBS
MES
12
10
8
6
4
2
0
D
Δcda1::HA75ScCda1
Acceptor
AFP1550
Donor
HA75ScCda1
Merge
Pre
Pre
Post
ns
13
13
FRET Efficiency (%)
ScCda1
WGA
+ AFP1
E
AD
BD
LW
LWAH
–
ScCda1
ScCda2
–
AFP1
ScCda1
AFP1
ScCda2
AFP1
–

**FIG S8**. AFP1 extends the inhibitory effect towards *C. graminicola* and *S. cerevisiae.*

(A, B) *C. graminicola* and *S. cerevisiae* cells were treated with AFP1 and AFP1* proteins, followed by immunostaining to detect the localization of AFP1. Bars:10 μm. (A, E) Yeast two-hybrid assays to detect the interactions of AFP1 and CDA proteins of *C. graminicola* (A) or *S. cerevisiae* (E). Yeast transformants containing two plasmids expressing the indicated proteins fusion to the GAL4 activation domain (AD) or binding domain (BD) were grown on SD-LW for growth control and on SD-LWHA or LWH plates containing 10 mM of 3AT to assess protein interaction. –, empty vector. Self-interaction of chorismate mutase (Cmu1) served as a positive control, and AD-AFP1/BD and AD/BD-CDA were used as negative controls. Similar results were observed in at least two independent experiments. (C) qRT-PCR analysis of *S. cerevisiae CDA* genes. Total RNA extracted from BY4742 cells after the 4 hour-incubation in YPD medium, 1xPBS buffer (pH7.2), and 10 mM MES (pH5.5) buffer was subjected to qRT-PCR analysis. Expression levels of *CDA* genes are normalized to the *ACTIN* gene and relative to the expression level of *CDA* in YPD, which is set to 1. Data represent the mean ± sd of the three biological replicates. (D) *S. cerevisiae* cells Δ*cda1*::$HA_{75}$ScCda1 constitutively expressing $HA_{75}$ScCda1 from the *gpd* promoter were immunostained to localize ScCda1 or stained with WGA-AF488 to locate chitin, prior to an incubation with DyLight 550 labeled AFP1 ($AFP1^{550}$). The number of spots from three independent experiments used in the analysis is indicated above columns. Values represent the mean ± sd. Representative images of cells displaying fluorescence of $AFP1^{550}$ and $HA_{75}$ScCda1/WGA-AF488 taken before and after photobleaching $AFP1^{550}$ were shown. ns, no significant differences in FRET efficiency between $HA_{75}$ScCda1 and WGA488-strained chitin determined by a two-tailed Student's *t*-test.
