# Supplementary material for: Maize Antifungal Protein AFP1 Elevates Fungal Chitin Levels by Targeting Chitin Deacetylases and Other Glycoproteins
Source: mBio. 2023 Mar 22;14(2):e00093-23. doi: 10.1128/mbio.00093-23 (PMC10128019; doi:10.1128/mbio.00093-23)
Supplement: TABLE S1 [file mbio.00093-23-s0009.pdf]

TABLE S1 Strains, plasmids and oligonucleotides used in this study

Table S1A Strains used in this study

**Strain**	**Genotype**	**Resistance***	**References**
SG200	*a1 mfa2 bW2 bE1, ble;*	P	(1)
SG200::GFP	*a1 mfa2 bW2 bE1, ble; ip^R[p123]ip^S;*	P, C	This work
SG200Δpmt4	*a1 mfa2 bW2 bE1, ble; umag05433(pmt4)::cbx*	P, C	(2)
SG200Δcda2,3,4,5,6^{em},Δ7 (SG200Δcda2-7)	*a1 mfa2 bW2 bE1, ble; cda2Δ121 - 134; cda3Δ21 - 28; cda4Δ146 - 155; cda5 Δ101 -113; cda6 Δ74 - 83; umag02381(cda7)::hyg*	P, HY	(3)
SG200Δcda1,3,4,5,6^{em} (SG200Δcda1,3-6)	*a1 mfa2 bW2 bE1, ble; cda1Δ121-127; cda3Δ21-28; cda4Δ146-155; cda5Δ101-113; cda6Δ74-83;*	P	(3)
SG200Δcda1,3,4,5,6^{em},Δ7 (SG200Δcda1,3-7)	*a1 mfa2 bW2 bE1, ble; cda1Δ121-127; cda3Δ21-28; cda4Δ146-155; cda5Δ101-113; cda6Δ74-83; umag02381(cda7)::hyg*	P, HY	This study
SG200::Cmu1HA	*a1 mfa2 bW2 bE1, ble; ip^r[P_{otef}-cmu1-HA]ip^s*	P, C	(4)
FB1	*a1b1*		(5)
FB2	*a2b2*		(5)
SG200Δcda1	*a1 mfa2 bW2 bE1, ble; umag00638(cda1)::hyg*	P, HY	This work
SG200Δcda2	*a1 mfa2 bW2 bE1, ble; umag01143(cda2)::hyg*	P, HY	This work
SG200Δcda7	*a1 mfa2 bW2 bE1, ble; umag02381(cda7)::hyg*	P, HY	This work
SG200Δcda1::HA_{76}Cda1	*a1 mfa2 bW2 bE1, ble; cda1::hyg; ip^R[P_{otef}-HA_{76}cda1]ip^S*	P, HY, C	This work
SG200Δcda2::HA_{118}Cda2	*a1 mfa2 bW2 bE1, ble; cda2::hyg; ip^R[P_{otef}-HA_{118}cda2]ip^S*	P, HY, C	This work
SG200::HACda1	*a1 mfa2 bW2 bE1, ble; ip^r[P_{otef}-HAcda1]ip^s*	P, C	This work
SG200Δcda1,3-6::HA_{76}Cda1	*a1 mfa2 bW2 bE1, ble; cda1Δ121-127; cda3Δ21-28; cda4Δ146-155; cda5Δ101-113; cda6Δ74-83; ip^R[P_{otef} -HA_{76}cda1]ip^S;*	P, C	This work
SG200Δcda1,3-6:: Cda1Strep(ΔGPI)	*a1 mfa2 bW2 bE1, ble; cda1Δ121-127; cda3Δ21-28; cda4Δ146-155; cda5Δ101-113; cda6Δ74-83; ip^R[P_{act}-cda1-Strep(ΔGPI)]ip^S;*	P, C	This work
SG200Δcda2-7:: Cda2Strep(ΔGPI)	*a1 mfa2 bW2 bE1, ble; cda2Δ121-134; cda3Δ21-28; cda4Δ146-155; cda5 Δ101-113; cda6 Δ74-83; cda7::hyg; ip^R[P_{act}-cda2-Strep(ΔGPI)]ip^S;*	P, HY, C	This work
SG200Δcda1,3-6:: Cda3Strep(ΔGPI)	*a1 mfa2 bW2 bE1, ble; cda1Δ121-127; cda3Δ21-28; cda4Δ146-155; cda5Δ101-113; cda6Δ74-83; ip^R[P_{act}-cda3-Strep(ΔGPI)]ip^S;*	P, C	This work
SG200Δcda1,3-6:: Cda5Strep(ΔGPI)	*a1 mfa2 bW2 bE1, ble; cda1Δ121-127; cda3Δ21-28; cda4Δ146-155; cda5Δ101-113; cda6Δ74-83; ip^R[P_{otef}-cda5-Strep(ΔGPI)]ip^S;*	P, C	This work
SG200Δcda2-7:: Cda7Strep(ΔGPI)	*a1 mfa2 bW2 bE1, ble; cda2Δ121-134; cda3Δ21-28; cda4Δ146-155; cda5 Δ101-113; cda6 Δ74-83;cda7:: hyg; ip^R[P_{otef}-cda7-Strep(ΔGPI)]ip^S;*	P, HY, C	This work
SG200Δcda1,3-6::Cda4HA	*a1 mfa2 bW2 bE1, ble; cda1Δ121-127; cda3Δ21-28; cda4Δ146-155; cda5Δ101-113; cda6Δ74-83; ip^R[P_{cda4}-cda4HA]ip^S;*	P, C	This work
SG200Δpmt4::HA_{76}Cda1 (mig2-6 locus)	*a1 mfa2 bW2 bE1, ble; um05433::cbx; [P_{otef} -HA_{76}cda1]mig2-6;*	P, C, G	This work
SG200Δpmt4::HA_{118}Cda2 (mig2-6 locus)	*a1 mfa2 bW2 bE1, ble; um05433::cbx; [P_{otef} -HA_{118}cda2]mig2-6;*	P, C, G	This work
SG200Δpmt4::Cda4HA (mig2-6 locus)	*a1 mfa2 bW2 bE1, ble; um05433::cbx; [P_{otef} -cda4HA]mig2-6;*	P, C, G	This work
SG200::HA_{76}Cda1	*a1 mfa2 bW2 bE1, ble; ip^R[P_{otef}- HA_{76}cda1] ip^S;*	P, C	This work
SG200::HA_{118}Cda2	*a1 mfa2 bW2 bE1, ble; ip^R[P_{otef}- HA_{118}cda2] ip^S;*	P, C	This work
SG200::Cda4HA (mig2-6 locus)	*a1 mfa2 bW2 bE1, ble;[P_{otef}-cda4HA] mig2-6;*	P, G	This work
SG200::1204Strep	*a1 mfa2 bW2 bE1, ble; ip^R[P_{otef}- 1204Strep] ip^S;*	P, C	This work
SG200Δcda2-7::HA_{118}Cda2	*a1 mfa2 bW2 bE1, ble; cda2Δ121-134; cda3Δ21-28; cda4Δ146-155; cda5 Δ101-113; cda6 Δ74-83; cda7::hyg; ip^R[P_{otef}-HA_{118}cda2]ip^S;*	P, HY, C	This work
SG200Δpmt4::Cda1Strep(Δ GPI)	*a1 mfa2 bW2 bE1, ble; um05433::cbx; [P_{act}-CDA1-Strep(ΔGPI)]mig2-6;*	P, C, G	This work
SG200Δcda1,3-7:: GFP_{63}cda7	*a1 mfa2 bW2 bE1, ble; cda2Δ121-134; cda3Δ21-28; cda4Δ146-155; cda5 Δ101-113; cda6 Δ74-83; cda7:: hyg; ip^R[P_{otef}-gfp_{63}cda7]ip^S;*	P, HY, C	This work

SG200Δcda7::GFP_{63}cda7	*a1 mfa2 bW2 bE1, ble; cda7:: hyg; ip^R[P_{otef}-gfp$_{63}$cda7]ip^S;*	P, HY, C	This work
SG200Δcda1::NodB-HA	*a1 mfa2 bW2 bE1, ble; cda1::hyg; ip^R[P_{otef}-NodB-HA]ip^S*	P, HY, C	This work
BY4742	MATα *his3*Δ1 *leu2*Δ0 *lys2*Δ0 *ura3*Δ0		(6)
BY4742Δcda1	MATα *his3*Δ1 *leu2*Δ0 *lys2*Δ0 *ura3*Δ0 cda1Δ	G418	(6)
BY4742Δcda1,2	MATα *his3*Δ1 *leu2*Δ0 *lys2*Δ0 *ura3*Δ0 cda1Δ cda2Δ::*HIS3MX6*	G418, His	This work
BY4742Δcda1:: mCherryHA_{75}ScCda1	MATα *his3*Δ1 *leu2*Δ0 *lys2*Δ0 *ura3*Δ0 *cda1*Δ:: *HIS3MX6, P_{native}-mCherryHA$_{75}$ScCDA1*(*YLR307W*)	G418, His	This work
BY4742Δcda1 HA_{75}ScCda1	MATα *his3*Δ1 *leu2*Δ0 *lys2*Δ0 *ura3*Δ0 *cda1*Δ:: *HIS3MX6, P_{gpd}-HA$_{75}$ScCDA1*(*YLR307W*)	G418, His	This work

* Phleomycin (P), Hygromycin (HY), Carboxin (C), Geneticin (G), Histidine (His)

Table S1B Plasmids used in this study

Plasmids	Description	Reference
p123	Plasmid containing the *gfp* gene controlled by constitutive promoter *otef, nos* terminator, the *U. maydis* carboxin resistant *ip* allele (ip^R), and ampicillin resistance gene. This plasmid served as backbone to insert gene of interest ectopically into the *U. maydis ip* locus.	(7)
pHwtFRT	Plasmid containing the hygromycin resistance cassette (Hyg^R).	(8)
pJET-cda1KO	The left and right borders of cda1 gene were amplified from SG200 gDNA using the primer pairs #608/609 and #610/611. Fragment containing hygromycin resistance cassette was obtained from SfiI digestion of pHwtFRT. The three DNA fragments were integrated into EcoRV digested pJET plasmid via Gibson assembly.	This study
pJET-cda2KO	The left and right borders of *cda2* gene were amplified from SG200 gDNA using the primer pairs #612/613 and #614/615. The hygromycin resistance cassette was obtained from SfiI digestion of pHwtFRT. The three DNA fragments were integrated into EcoRV digested pJET plasmid via Gibson assembly.	This study
pJET-cda7KO	The left and right borders of cda7 gene were amplified from SG200 gDNA using the primer pairs #563/564 and #565/566. The hygromycin resistance cassette was obtained from SfiI digestion of pHwtFRT. The three DNA fragments were integrated into the EcoRV digested pJET plasmid via Gibson assembly.	This study
P_{act}-mcherry	p123-derived plasmid containing *mcherry* gene under the control of *actin* promoter (UMAG_11232).	(9)
P_{otef}-vp1HA	p123-derived plasmid used in constitutively expressing secreted Vp1HA in *U. maydis*	(10)
P_{otef}-rsp3-HA (mig2-6 locus)	Plasmid containing mig2-6 locus geneticin resistance cassette (G418)	Ma et al, unpublished
P_{otef}-1204-Strep	A PCR fragment amplified from SG200 gDNA using the primer pairs #65/102 was digested with XmaI/XbaI and ligated into the XmaI/XbaI digested p123 plasmid.	
P_{act}-cda1-Strep (ΔGPI)	Two PCR fragments amplified from SG200 gDNA (the primer pair #409/410) and from p123 plasmid (primer pair #76/411) were integrated into the NcoI/EcoRV digested P_{act}-mcherry via Gibson assembly.	This study
P_{act}-cda2-Strep (ΔGPI)	XmaI/XbaI digested PCR fragment containing *cda2* gene amplified using the primer pairs #453/454 from SG200 gDNA, and XbaI/EcoRV digested fragment containing StrepII from P_{act}-cda1strep (ΔGPI) were ligated into XmaI/EcoRV digested P_{act}-mcherry vector.	This study
P_{act}-cda3-Strep (ΔGPI)	XmaI/XbaI digested PCR fragment amplified from SG200 gDNA with the primer pairs #455/456 was ligated into the XmaI/XbaI digested Pact-cda2Strep (ΔGPI) plasmid.	This study
P_{otef}-cda5-Strep (ΔGPI)	XmaI/XbaI digested PCR fragment amplified from SG200 gDNA using primer pair #436/473 and XbaI/EcoRV digested fragment from P_{act}-cda2Strep (ΔGPI) were ligated into the XmaI/EcoRV digested p123 plasmid.	This study
P_{otef}-cda7-Strep (ΔGPI)	XmaI/XbaI digested PCR fragment amplified from SG200 gDNA using primer pair #438/457 was ligated into the XmaI/XbaI digested P_{otef}-cda5Strep (ΔGPI) plasmid.	This study
P_{cda4}-cda4HA	KpnI/XbaI digested PCR fragment containing the native promoter and ORF of *cda4* gene amplified from SG200 gDNA using primer pair #1/2 was ligated into the KpnI/XbaI digested P_{otef}-vp1HA backbone.	This study
P_{otef}-HAcda1	Two PCR fragments amplified using the primer pairs #358/370 and #368/369 from SG200 gDNA, were integrated into the BamHI/NotI digested p123 vector via Gibson assembly.	This study
P_{otef}-HA_{76}cda1	Two fragments of *cda1* amplified using the primer pairs #358/391 and #368/369 from SG200 gDNA, were integrated into BamHI/NotI digested p123 vector via Gibson assembly.	This study
P_{otef}-HA_{118}cda2	Two fragments of *cda2* amplified using the primer pairs #453/5 and #3/4 from SG200 gDNA were digested with XmaI/NgoMIV and NgoMIV/NotI respectively and ligated into the XmaI/NotI digested p123 vector.	This study
P_{otef}-HA_{63}cda7	Two *cda7* fragments amplified using the primer pairs #648/649 and #650/651 from SG200 gDNA, were integrated into XmaI/NotI digested p123 vector via Gibson assembly.	This study
P_{otef}-gfp_{63}cda7	Two fragments of *cda7* amplified using the primer pairs #21/779 and #782/651 from P_{otef}_HA_{63}cda7 and one fragment of *gfp* amplified using primer pair #780/781 from p123 were integrated into XmaI/Not-digested p123 vector via Gibson assembly.	This study
P_{otef}-HA_{76}cda1 (mig2-6 locus)	SbfI/NotI-digested *HA_{76}cda1* fragment from P_{otef} -HA_{76}cda1 was ligated into SbfI/NotI-digested P_{otef}-rsp3-HA (mig2-6)	This study
P_{otef}-HA_{118}cda2 (mig2-6 locus)	SbfI/NotI-digested *HA_{118}cda2* from P_{otef} -HA_{118}cda2 was ligated into the SbfI/NotI digested P_{otef}-rsp3-HA (mig2-6)	This study
P_{otef}-cda4HA (mig2-6 locus)	SbfI/NotI-digested *cda4HA* from P_{otef} -cda4HA was ligated into SbfI/NotI-digested P_{otef}-rsp3-HA (mig2-6)	This study

P_{otef}-NodB-HA	Two fragments of *cda1* amplified from plasmid P_{otef}-HAcda1using primer pairs #12/13 and #14/15, were integrated into BamHI/XbaI-digested p123 vector via Gibson assembly.	This study
**Yeast two-hybrid and yeast plasmid constructs**		
pGADT7	Yeast expression vector that is designed to express a protein of interest fused to a GAL4 activation domain	Clontech
pGBKT7	Yeast expression vector that is designed to express a protein of interest fused to a GAL4 DNA binding domain	Clontech
pAD-cmu1/pBD_cmu1	A NdeI/EcoRI digested PCR fragment amplified from SG200 gDNA using primer pair #354/355 was ligated into the NdeI/EcoRI digested pGADT7 and pGBKT7 vectors.	This study
pAD-AFP1/BD-AFP1	A NdeI/BamHI digested-PCR fragment amplified from maize cDNA using primer pair #288/289 was ligated into the NdeI/BamHI digested pGADT7 and pGBKT7 vectors.	This study
pBD-cda1	A NdeI/EcoRI digested-PCR fragment amplified from SG200 gDNA using primer pair #352/356 was ligated into the NdeI/EcoRI digested pGBKT7 vector.	This study
pBD-cda2	A NdeI/BamHI digested-PCR fragment amplified from SG200 gDNA using primer pair #374/375 was ligated into the NdeI/BamHI digested pGBKT7 vector.	This study
pBD-cda3	A NdeI/BamHI digested-PCR fragment amplified from SG200 gDNA using primer pair #376/377 was ligated into the NdeI/BamHI digested pGBKT7 vector.	This study
pBD-cda4	A NdeI/EcoRI digested-PCR fragment amplified from SG200 gDNA using primer pair #378/379 was ligated into the NdeI/EcoRI digested pGBKT7 vector.	This study
pBD-cda5	A NdeI/BamHI digested-PCR fragment amplified from SG200 gDNA using primer pair #380/381 was ligated into the NdeI/BamHI digested pGBKT7 vector.	This study
pBD-cda7	A NdeI/EcoRI digested-PCR fragment amplified from SG200 gDNA using primer pair #401/402 was ligated into the NdeI/EcoRI digested pGBKT7 vector.	This study
pBD-cts1	A NdeI/BamHI digested-PCR fragment amplified from SG200 gDNA using primer pair #290/291 was ligated into the NdeI/BamHI digested pGBKT7 vector.	This study
pBD-cts2	A NdeI/BamHI digested-PCR fragment amplified from SG200 gDNA using primer pair #292/293 was ligated into the NdeI/BamHI digested pGBKT7 vector.	This study
pBD-cts3	A NdeI/BamHI digested-PCR fragment amplified from SG200 gDNA using primer pair #294/295 was ligated into the NdeI/BamHI digested pGBKT7 vector.	This study
pBD-cts4	A SfiI/XmaI digested-PCR fragment amplified from SG200 gDNA using primer pair #296/297 was ligated into the SfiI/XmaI digested pGBKT7 vector.	This study
pBD-Sccda1	A NcoI/NotI digested-PCR fragment amplified from AH109 cDNA using primer pair #474/475 was ligated into the NcoI/NotI digested pGBKT7 vector.	This study
pBD-Sccda2	A NcoI/NotI digested-PCR fragment amplified from AH109 cDNA using primer pair #476/477 was ligated into the NcoI/NotI digested pGBKT7 vector.	This study
pBD-Cg386	A NdeI/EcoRI digested-PCR fragment amplified from *C. graminicola* CgM2 cDNA using primer pair #488/489 was ligated into the NdeI/EcoRI digested pGBKT7 vector.	This study
pBD-Cg7915	Two PCR fragments amplified from *C. graminicola* CgM2 cDNA using the primer pairs #490/559 and #560/491 were combined and performed the overlap extension PCR. The overlap extension PCR fragment was digested with NdeI and EcoRI, and ligated into the NdeI/EcoRI digested pGBKT7 vector.	This study
pAD-chs5	A SfiI/BamHI digested-PCR fragment amplified from SG200 using primer pair #323/324 was ligated into the SfiI/BamHI digested pGADT7 vector.	This study
pAD-chs6	A SfiI/XmaI digested-PCR fragment amplified from SG200 using primer pair #325/326 was ligated into the SfiI/XmaI digested pGADT7 vector.	This study
pAD-chs7	A NdeI/SacI digested-PCR fragment amplified from SG200 using primer pair #327/328 was ligated into the NdeI/SacI digested pGADT7 vector.	This study
p425 GPD	Expression vector (ATCC# 87359)	(11)
P_{native}-mCherryHA$_{75}$ScCda1	Two *cda1* fragments amplified from BY4742 gDNA using the primer pairs #6/7 and #8/9, and mCherryHA fragment amplified using primer pair #10/11, combined and integrated into SacI/SalI-digested p425-GPD vector via Gibson assembly	This study
p425-HA$_{75}$ScCda1	Two PCR fragments amplified from plasmid P_{native}-mCherryHA75-ScCda1 using primer pairs #18/#19 and #16/17 were integrated into SpeI/SalI-digested p425-GPD via Gibson assembly	This study

Table S1C Oligonucleotides used in this study

Oligonucleotides for plasmid construction	
**Name**	**Sequence (5' to 3')**
#1	CTCGGTACCATCGCGGTTGTCGTCGTACT
#2	GGTCTAGAGTTAGCCGCTCCAGGAGC
#3	CCTGCCGGCTACCCCTACGACGTGCCCGACTATGCCACCGACATGACCAACGTCTG
#4	TAAGCGGCCGCTCAGAGCAAGAGAGCAAAAGCG
#5	TAGCCGGCAGGGTAGACGGTG
#6	GGGAACAAAAGCTGGAGCTCAGAAGACTGGTCGCCAGCTC
#7	CTTGCTCACTAATTCTGGCACTTCAGTAAG
#8	AGATTACGCTGATAGGTACTATCCTGGCCAGTGTC
#9	TTACATGACTCGAGGTCGACCTAGTCGTAGCGTTCGATG
#10	TGCCAGAATTAGTGAGCAAGGGCGAGGAGG
#11	AGTACCTATCAGCGTAATCTGGAACATCG
#12	CCGATATAGTTTGCAGGTGG
#13	AAAGCCGATGGTCATGTGGCTAGCAAGAGCGCTGCCAGCTAG
#14	CTTGCTAGCCACATGACCATCGGC
#15	GGACGTCGTAGGGATATCTAGAGTCTACAATCCAGCCAAG
#16	TTACATGACTCGAGGTCGACCTAGTCGTAGCGTTCGATG
#17	TACCCATACGATGTTCCAGATTAC
#18	TCTGGAACATCGTATGGGTAGCTCACTAATTCTGGCACTTC
#19	TAGTTTCGACGGATCTAGAACTAGTATGAAAATTTTCAATACAATAC
#21	GCTTAACTATGCGGCATCAG
#65	ATATCTAGAGTTGGGCGAGACGTTCTC
#76	CCTTTGAGTGAGCTGATACC
#102	CCCCCGGGATGAAAGTCACATCTGTGATCG
#288	CTGCATATGGCGGACTCCATCGGCAGCTAC
#289	GACGGATCCTTAAGGACGCACGACGATCTTG
#290	GCTCATATGTTTGGACGTCTTAAGCACAGG
#291	GATGGATCCTACTTGAGGCCGTTCTTG
#292	GCTCATATGGTGCCTCACGAGCAGAGC
#293	GATGGATCCTAGCTCAATCCGGCAGCGTC
#294	GCTCATATGGCGCTGAACAATGATGGATC
#295	GTGGAATTCCTAGGAAGAGATAGCACCTG
#296	TATGGCCATGGAGGCCCTTTGGCCTCACCCAACCAC
#297	CCACCCGGGCTAATGCATGTTGCACATGCC
#323	TATGGCCATGGAGGCCAATCCTTTCGAATCTCTTCC
#324	GATGGATCCTCAGTCAAAGCTTGGAGGAG
#325	TATGGCCATGGAGGCCTCGACCAAAGACGCCCACG
#326	TTACCCGGGTCAGGCTTGTGCGCCACCGGAC
#327	GCTCATATGCCCGCAGTTGAGCGCAAC
#328	CTCGAGCTCAACTGAATCGATCATGATAG
#352	GCTCATATGGGCGACTTCACCGTCAAGATCC
#354	GCTCATATGGCTGTATCTGGCAAGTCG
#355	GTGGAATTCTTAGGTGCACTTGTTGGCGTGG
#356	GTGGAATTCTCAAAGCAGAGTTGCCAC
#358	CACAGACAACATCATCCACGGGATCCATGCTGCGTTTGCTACTTC
#368	TACCCATACGACGTACC
#369	CGATCTGCAGCCGGGCGGCCGCTCAAAGCAGAGTTGCCAC
#370	TCTGGTACGTCGTATGGGTAAGCAAGAGCGCTGCCAGCTA
#374	GCTCATATGGCTGGACGCCACGAGCGAGGCCTTGT
#375	GATGGATCCTCAGAGCAAGAGAGCAAAAGCG
#376	GCTCATATGAACATCGGCCGTGGCGTAC
#377	GATGGATCCTCAGGCGAGCATGGCGATGG
#378	GCTCATATGTCCCCCACTACAATGAGCAC
#379	GTGGAATTCTCAGTTAGCCGCTCCAGGAG
#380	GCTCATATGAGTCCCGACTTTGAGAAGCG
#381	GATGGATCCTTAGACGAGAAGACCGAAGAAG
#391	AGCGTAATCTGGTACGTCGTATGGGTAGCTGCCGCCTTGATTGAG
#401	GCTCATATGCATGGTGGCGACCTTCTC
#402	TGGAATTCTTAGATAAAGACCATCATAGCACCG
#409	AAAACCTAGTTCACAGTCATCCCATGCTGCGTTTGCTACTTC
#410	TTTTTCAAACTGCGGATGTGACCATCTAGAGCTGGTGGCGATGCCCTGTTTG
#411	TGGTCACATCCGCAGTTTGAAAAATAGAGCGGCCGCCCGGCTGCA

#436	TTTTCAAACTGCGGATGTGACCATCTAGAGGTGCCAGCACCCCCAGTC
#438	TTTTTCAAACTGCGGATGTGACCATCTAGAGCTGCCGAACGTCCTGTAG
#453	CATCCCGGGATGCGTCTCTCCGTCTCCG
#454	ACCATCTAGAACTCGACTGACTCTTG
#455	CATCCCGGGATGAAGCTTTCCTCGACAGC
#456	CCATCTAGACTTATCTGCGGACTG
#457	CATCCCGGGATGAAGTCTACCACGGTATTCTCG
#473	CATCCCGGGATGGTCAAAATCACTTTTGC
#474	TGGCCATGGAATCAAATGGGAGTACCGCATTGATGGG
#475	TATGCGGCCGCCTAGTCGTAGCGTTCGATG
#476	TGGCCATGGCCGAAGCTAATAGGGAAGATTTAAAG
#477	GCCGCGGCCGCTTAGGACAAGAATTCTTTTATG
#488	GCTCATATGGGCCCGGTCACCCGCCGTCAAAG
#489	GGGAATTCTTAGGACTTGTACCAGTTCTC
#490	CGCCATATGACTCCTTTCCGCCGCCAAAC
#491	GCCGAATTCTCAGGCACAGGTACCGAAAAG
#563	GCTCGAGTTTTTCAGCAAGATAATATTGTTGCGCTCATGAGCCCTTG
#564	AGAATAGGAACTTCTGGCCATCTAGGCCCGTGATGGTTCACGCCAAACG
#565	AGTATAGGAACTTCTGGCCTGAGTGGCCATATGCTACGTCGTATCTCGCACTTC
#566	AGGAGATCTTCTAGAAAGATAATATTCGACCTAAAAAATGGCGCTGCTGC
#559	CGGTGTTTTGGTGAACATCGTGGGCCAGAGCGATG
#560	TCTGGCCCACGATGTTCACCAAAACACCGCCGAGT
#608	GCTCGAGTTTTTCAGCAAGATAATATTGCACGAGTATATTTTCGAGAGG
#609	AGAATAGGAACTTCTGGCCATCTAGGCCGTCGGAAAAAAAGCGATCTCTC
#610	AGTATAGGAACTTCTGGCCTGAGTGGCCACTTAGCATTGCCAACTGCATTC
#611	AGGAGATCTTCTAGAAAGATAATATTGCAGATGTTGATTCTTTGAGG
#612	GCTCGAGTTTTTCAGCAAGATAATATTCTTGGTTTGCCTCAGATGTTC
#613	AGAATAGGAACTTCTGGCCATCTAGGCCGATATGAAAAGTCGATGAGGG
#614	AGTATAGGAACTTCTGGCCTGAGTGGCCGCTTCACCATACGGCTCAGC
#615	AGGAGATCTTCTAGAAAGATAATATTGCAGCGCAACTCGCACCATAC
#648	AACATCATCCACGGGATCCCCCGGGATGAAGTCTACCACGGTATTCTC
#649	ATCTGGTACGTCGTATGGGTAAGCGGAAGCGCCACCACCA
#650	TACCCATACGACGTACCAGATTATGCTGCTCGATACAGCTGCGACC
#651	ACGATCTGCAGCCGGGCGGCCGCTTAGATAAAGACCATCATAGCACCG
#779	AGCGGAAGCGCCACCACCAG
#780	CTGGTGGTGGCGCTTCCGCTATGGTGAGCAAGGGCGAGGA
#781	GGGTCGCAGCTGTATCGAGCCTTGTACAGCTCGTCCATGC
#782	GCTCGATACAGCTGCGACCC
**Oligonucleotides for RT-PCR**	
ppi-F	ACATCGTCAAGGCTATCG
ppi-R	AAAGAACACCGGACTTGG
cda1F (UMAG_00638)	CGAGTACAAGTGCTACTACC
cda1R	AGGCCGAAGGTAGCAGGAAC
cda2F (UMAG_01143)	ACCAGAACGCGTGGTTCATC
cda2R	CGACAGCCAAAGCGCCAAAG
cda3F (UMAG_11922)	CCACGGGTACCAACACCAAC
cda3R	ATGGCCGAGAGCAAGACGAC
cda4F (UMAG_01788)	CAACCACGAGACCGTCCAAG
cda4R	CTGCAGGTCCAGGTGGAATC
cda5F (UMAG_02019)	TTCCCGCCGCTGCTCAAATG
cda5R	TTGGTGGAAGTGAGCTGATG
cda6F (UMAG_05792)	TGGCTCTGGGAACCGTCAAG
cda6R	TACCCATCACGGTGGCAACAG
cda7F (UMAG_02381)	ACTTGTGCGTTTGACGACTC
cda7R	ACACGGTCCAGCCAATCAAG
GLRG_07915_F	TCTGGCCCACGATGTTCAC
GLRG_07915_R	TAGGAGTAGCGGTGCTAGTG
GLRG_11148_F	CAATTGCTGCTCTCACAGGTC
GLRG_11148_R	TAGAAGCCGAGCCGGATGTAG
GLRG_11238_F	TGCTGTTCCAGTGCGGGATGGTG
GLRG_11238_R	AGCCTTGGCATTGTACGCCTTG
GLRG_11241_F	TCGTCAAACGAGCCGAGTG
GLRG_11241_R	GGTGGTAATTATTGAGCCATAAG
GLRG_09099_F	CGAGCAGTACTTGAGGAAC
GLRG_09099_R	GTATGGGAAGTGGGATCTC
GLRG_00386_F	CGTCACCGACCTCACTAAC

GLRG_00386_R	ATCTTCTGCACGAGGGAGATG
GLRG_03854_F	GGCTTCGTACACTGACGACTTG
GLRG_03854_R	GCCGAGGATGTTGCGAATAGCC
GLRG_02208_F	TTGCTGCTCCTCGTACGGATG
GLRG_02208_R	CCGGCAACAGCAGTCGACAAACAG
Actin F (GLRG_05255)	GTGGCAGCACTCTGTACAAG
Actin R (GLRG_05255)	GCCGTATTCCTCGTACTCAG
ScActin F	AGAGTTGCCCCAGAAGAACA
ScActin R	GGCTTGGATGGAAACGTAGA
ScCda1_F	GGTACACACACGTGGTCACA
ScCda1_R	ACAAGACAACCGTTAGGCCA
ScCda2_F	AAGGGCAGCTACAAAAACGC
ScCda2_R	AAAGAGCACGAGTCCCTTGG
**Oligonucleotides for yeast mutant generation**	
BY4742Δcda1_F	CAAAAGAGTTGTTATTATTTCTACGGATCGGCAATTGAAACGGATCCCCGGGTTAATTAA
BY4742Δcda1_R	GTAATTTAATTCTTCCTTATTTTCTTCAATTCCCTGAAAAGAATTCGAGCTCGTTTAAAC

References

1. Kamper J, Kahmann R, Bolker M, Ma LJ, Brefort T, Saville BJ, Banuett F, Kronstad JW, Gold SE, Muller O, Perlin MH, Wosten HA, de Vries R, Ruiz-Herrera J, Reynaga-Pena CG, Snetselaar K, McCann M, Perez-Martin J, Feldbrugge M, Basse CW, Steinberg G, Ibeas JI, Holloman W, Guzman P, Farman M, Stajich JE, Sentandreu R, Gonzalez-Prieto JM, Kennell JC, Molina L, Schirawski J, Mendoza-Mendoza A, Greilinger D, Munch K, Rossel N, Scherer M, Vranes M, Ladendorf O, Vincon V, Fuchs U, Sandrock B, Meng S, Ho EC, Cahill MJ, Boyce KJ, Klose J, Klosterman SJ, Deelstra HJ, Ortiz-Castellanos L, Li W, et al. 2006. Insights from the genome of the biotrophic fungal plant pathogen Ustilago maydis. Nature 444:97-101.
2. Fernandez-Alvarez A, Elias-Villalobos A, Ibeas JI. 2009. The O-mannosyltransferase PMT4 is essential for normal appressorium formation and penetration in Ustilago maydis. Plant Cell 21:3397-412.
3. Rizzi YS, Happel P, Lenz S, Urs MJ, Bonin M, Cord-Landwehr S, Singh R, Moerschbacher BM, Kahmann R. 2021. Chitosan and Chitin Deacetylase Activity Are Necessary for Development and Virulence of Ustilago maydis. mBio 12.
4. Djamei A, Schipper K, Rabe F, Ghosh A, Vincon V, Kahnt J, Osorio S, Tohge T, Fernie AR, Feussner I, Feussner K, Meinicke P, Stierhof YD, Schwarz H, Macek B, Mann M, Kahmann R. 2011. Metabolic priming by a secreted fungal effector. Nature 478:395-8.
5. Banuett F, Herskowitz I. 1989. Different a alleles of Ustilago maydis are necessary for maintenance of filamentous growth but not for meiosis. Proc Natl Acad Sci U S A 86:5878-82.
6. Brachmann CB, Davies A, Cost GJ, Caputo E, Li J, Hieter P, Boeke JD. 1998. Designer deletion strains derived from Saccharomyces cerevisiae S288C: a useful set of strains and plasmids for PCR-mediated gene disruption and other applications. Yeast 14:115-32.
7. Aichinger C, Hansson K, Eichhorn H, Lessing F, Mannhaupt G, Mewes W, Kahmann R. 2003. Identification of plant-regulated genes in Ustilago maydis by enhancer-trapping mutagenesis. Mol Genet Genomics 270:303-14.
8. Khrunyk Y, Munch K, Schipper K, Lupas AN, Kahmann R. 2010. The use of FLP-mediated recombination for the functional analysis of an effector gene family in the biotrophic smut fungus Ustilago maydis. New Phytol 187:957-968.
9. Lanver D, Muller AN, Happel P, Schweizer G, Haas FB, Franitza M, Pellegrin C, Reissmann S, Altmuller J, Rensing SA, Kahmann R. 2018. The Biotrophic Development of Ustilago maydis Studied by RNA-Seq Analysis. Plant Cell 30:300-323.

10. Cuong V. Hoang CKB, Lay-Sun Ma. 2021. A Novel Core Effector Vp1 Promotes Fungal Colonization and Virulence of *Ustilago maydis*. J of Fungi 7.
11. Mumberg D, Muller R, Funk M. 1995. Yeast vectors for the controlled expression of heterologous proteins in different genetic backgrounds. Gene 156:119-22.
